# CCL20-CCR6 axis mediates mucosal-associated invariant T-M2 macrophage crosstalk to drive immune dysregulation in liver cirrhosis

**DOI:** 10.3389/fimmu.2026.1859932

**Published:** 2026-07-20

**Authors:** Bochen Chen, Ruoling Li, Shuya Zhang, Yiting Lou, Shuo Zhang, Jiaheng Lou, Jingcheng Zhang, Chuyun Xu, Tao Jiang, Yuanlin Lv

**Affiliations:** 1 The First Affiliated Hospital of Zhejiang Chinese Medical University(Zhejiang Provincial Hospital of Chinese Medicine), Hangzhou, China; 2 Zhejiang Chinese Medical University School of Basic Medical Sciences, Hangzhou, China; 3 The Second Affiliated Hospital of Zhejiang Chinese Medical University, Hangzhou, China

**Keywords:** CCL20, cirrhosis, immune dysregulation, M2 macrophages, MAIT cells, single-cell transcriptomics

## Abstract

**Introduction:**

Cirrhosis is the end-stage of chronic liver disease characterized by progressive hepatic fibrosis and persistent immune dysregulation, yet the mechanisms by which immune cell crosstalk drives disease progression remain poorly understood.

**Methods:**

In the present study, we integrated bulk and single-cell transcriptomic analyses with immunofluorescence validation to investigate the role of the CCL20-CCR6 axis in MAIT cells–macrophages interactions during cirrhosis progression.

**Results:**

We identified eight core cirrhosis-associated immune genes (*OGN, IL32, CCL19, PDGFA, CCL20, NTS, SEMA3C, HSPA2*), among which CCL20 was selectively enriched in mucosal-associated invariant T cells (MAIT) and closely associated with their activation- and exhaustion-related transcriptional profiles. Cell-cell communication analysis revealed that the CCL20-CCR6 axis may specifically mediate bidirectional interactions between MAIT cells and M2 macrophages, forming a disease-specific positive feedback loop within the cirrhotic microenvironment. Immunofluorescence staining further confirmed the enrichment and colocalization of CCL20, MAIT cells, and M2 macrophages in cirrhotic tissues.

**Discussion:**

Collectively, these findings support the CCL20-CCR6 axis as a key regulator of hepatic immune dysregulation and fibrotic progression in liver cirrhosis, highlighting its potential as both a biomarker of disease activity and a therapeutic target for cirrhosis intervention.

## 1 Introduction

Cirrhosis represents the end-stage of chronic liver disease, resulting from persistent insults such as viral infection, metabolic dysfunction, alcohol abuse, or cholestatic injury. It is characterized by progressive fibrosis, vascular remodeling, and eventual hepatic failure, accounting for over one million annual deaths globally (1, 2). Pathogenetically, cirrhosis involves excessive extracellular matrix (ECM) deposition, sustained activation of hepatic stellate cells (HSCs), and significant remodeling of the liver microenvironment, accompanied by persistent inflammation and immune dysregulation (3–5). Accumulating evidence implicates aberrant immune responses as a central mechanism in cirrhosis initiation and progression, wherein the interplay between innate and adaptive immune cells coordinately regulates fibrogenesis and hepatic dysfunction (6, 7).

In recent years, research has increasingly focused on the immune system in cirrhosis, particularly regarding intercellular communication, immune infiltration, and immunometabolic reprogramming. Dynamic changes in hepatic innate and adaptive immune cells are considered pivotal in the initiation and progression of fibrosis. Concurrently, the remodeling of cytokine and chemokine networks within the cirrhotic microenvironment perpetuates inflammation and sustains the activation of hepatic fibroblasts, thereby impeding fibrosis reversal (8). Studies have shown that certain non-conventional T cell populations, such as Mucosal-Associated Invariant T (MAIT) cells, exhibit altered frequencies and functions in liver disease and may contribute to cirrhosis pathogenesis (9).

MAIT cells are relatively enriched in the livers of healthy individuals, forming a major component of the liver’s atypical T-cell population. These cells express a range of chemokine receptors associated with liver retention and tissue-specific homing (including CCR6 and CXCR6), which enables their responsiveness to ligands expressed in the hepatic-biliary niche and facilitates their hepatic migration and localization (10). Evidence from multiple animal and human studies suggests that MAIT cells may contribute to the progression of liver fibrosis in cirrhosis by secreting proinflammatory cytokines, modulating the phenotype of macrophages and monocytes, and promoting the activation of hepatic stellate cells (HSCs). Conversely, functional inhibition of MAIT cells has been shown to partially reverse fibrosis in animal models, underscoring their plasticity and therapeutic potential in cirrhotic progression (11). Concurrently, the CCL20-CCR6 axis has been implicated in immune cell recruitment, inflammatory cascades, and liver necrosis-regeneration dynamics across various hepatic pathologies (12, 13). Further investigations have demonstrated that CCL20 specifically recruits CCR6-expressing immune cells—such as regulatory T cells (Tregs), subsets of Th17 and γδT cells, and certain myeloid lineages-via its exclusive high-affinity receptor CCR6 (14), thereby exerting a pivotal influence on both the inflammatory-fibrotic microenvironment and the tumor microenvironment in the liver. In the context of chronic liver injury and cirrhosis, activated stellate cells and inflammatory monocytes/macrophages secrete CCL20, perpetuating leukocyte infiltration and exacerbating fibrogenesis (15). Clinical and review studies have also reported a correlation between CCL20 levels and cirrhosis severity, suggesting its utility as a biomarker of disease activity and a potential therapeutic target for mitigating aberrant immune recruitment and subsequent fibrosis (2). In the hepatocellular carcinoma (HCC) microenvironment, tumor-associated macrophages or those undergoing lipid metabolic reprogramming markedly upregulate and secrete CCL20. This chemokine gradient facilitates the recruitment of CCR6^+^ Tregs, contributing to the establishment of an immunosuppressive milieu that supports tumor proliferation, invasion, and immune evasion (16). Thus, the CCL20-CCR6 axis functions not only as a critical chemotactic pathway directing immune cell trafficking in liver diseases, but also as a key nexus linking chronic inflammation, cirrhosis, and immune evasion in HCC, a connection warranting further exploration in the context of combined immuno-metabolic therapeutic strategies. Although prior studies have elucidated the proinflammatory contributions of MAIT cells to fibrogenesis and cirrhosis progression (9), the role of MAIT cells as both sources and responders within the CCL20-CCR6 axis remains poorly characterized. In particular, whether MAIT cells engage in reciprocal crosstalk with other hepatic immune subsets via this signaling pathway, and how this axis is mechanistically regulated during liver fibrosis, have yet to be systematically clarified.

Against this backdrop, we integrated multiple publicly available bulk transcriptomic datasets with immune-related gene sets. By applying differential expression analysis, weighted gene co-expression network analysis (WGCNA), and least absolute shrinkage and selection operator (LASSO) regression, we identified gene signatures closely associated with liver cirrhosis. Furthermore, single-cell transcriptomic analysis was performed to characterize MAIT cells subsets within the immune compartment, aiming to elucidate their functional roles in cirrhosis progression and their interactions with diverse immune cell populations. Particular emphasis was placed on the role of the CCL20-CCR6 signaling axis in MAIT cells.

## 2 Materials and methods

### 2.1 Data source

Raw transcriptomic data from patients with liver cirrhosis were obtained from the Gene Expression Omnibus (GEO) database (https://www.ncbi.nlm.nih.gov/gds), including GSE49541, GSE164760, and GSE14323. GSE49541 comprised 40 samples with fibrosis stage 0–1 and 32 samples with fibrosis stage 3-4 (17). Fibrosis stage 0–1 was defined as mild liver fibrosis, whereas fibrosis stage 3–4 was defined as cirrhosis. This dataset was designated as Dataset 1 for subsequent analyses.

GSE164760 included 6 healthy and 8 cirrhotic samples (18), while GSE14323 contained 19 healthy and 41 cirrhotic samples (19). These two datasets were merged and batch effects were corrected using the sva package (20), and the combined dataset was defined as Dataset 2 for further analysis. Detailed clinical metadata for all datasets are provided in **Supplementary Material 2**.

### 2.2 Identification of differentially expressed genes

In Dataset 1, differentially expressed genes (DEGs) were identified using the limma package (21). Genes with adjusted P value < 0.05 and an absolute log2 fold change (|log2FC|) ≥ 0.25 between mild liver fibrosis and cirrhosis were considered significantly differentially expressed.

### 2.3 Weighted gene co-expression network analysis

In Dataset 2, to reduce analytical noise and focus on genes with high biological variability, we calculated the median absolute deviation (MAD) of each gene across all samples, ranked genes in descending order of MAD, and selected the top 5,000 most variable genes for subsequent weighted gene co-expression network construction. The WGCNA package (22) was used to identify gene modules significantly associated with the clinical phenotype. We calculated the Pearson correlation between each module eigengene (ME) and the disease phenotype. The module with the high correlation coefficient (|r| ≥ 0.7) and statistically significant association with disease phenotype (adjusted P < 0.05) was defined as strong phenotype-associated module for subsequent core gene screening.

### 2.4 Machine learning

Immune-related genes (IRGs) were obtained from the ImmPort database (https://www.immport.org/home). Significantly upregulated differentially expressed genes (DEGs) identified in Dataset 1 were intersected with strongly positively correlated WGCNA modules (r ≥ 0.7, adjusted P < 0.05) and the IRG set. Similarly, significantly downregulated DEGs were intersected with strongly negatively correlated modules (r ≤ −0.7, adjusted P < 0.05) and the IRG set.Dataset 1 was used as the training set and Dataset 2 as the validation set. Candidate genes were subjected to least absolute shrinkage and selection operator (LASSO) regression using the glmnet package (23). The optimal penalty parameter (λ) was determined via 10-fold cross-validation, and the λ value corresponding to the minimum cross-validation classification error (lambda.min) was selected for final model fitting. Internal validation was performed in the training set, and external validation was conducted in the validation set to evaluate model performance. Genes with positive LASSO coefficients were defined as the LASSO gene signature for subsequent analyses. Based on the LASSO gene signature, single-sample gene set enrichment analysis (ssGSEA) was conducted in the validation set using the GSVA package (24). The resulting ssGSEA scores were compared between groups using the Wilcoxon rank-sum test to further assess the robustness of the signature.

### 2.5 Immune infiltration

In Dataset 2, immune cell infiltration was estimated using the Cell-type Identification by Estimating Relative Subsets of RNA Transcripts (CIBERSORT) algorithm implemented in the IOBR package (25). Additionally, ssGSEA was performed using 28 immune cell signature gene sets obtained from the TISIDB database (http://cis.hku.hk/TISIDB/). Differences in immune cell enrichment scores between healthy and cirrhotic samples were evaluated using the Wilcoxon rank-sum test. Correlation analysis was subsequently performed between ssGSEA scores derived from the LASSO gene signature and those based on the 28 immune cell signatures to identify immune cell types significantly and strongly associated with the LASSO signature.

### 2.6 Single-cell transcriptomic analysis

Single-cell RNA sequencing data (GSE181483) (26) were downloaded from GEO. Data processing was performed using the Seurat package with the following quality control criteria: nFeature_RNA > 200, nFeature_RNA < 7,000, and percent.mt < 15. Data normalization was conducted using the “LogNormalize” method, and the top 2,000 highly variable genes were retained. Batch effects were corrected using the Harmony package (27) with 40 dimensions.

After unsupervised clustering, cell type annotation was performed manually based on canonical cell-type-specific marker genes, with reference to human liver cell marker profiles from the CellMarker 2.0 database (http://117.50.127.228/CellMarker/). Major cell lineages were first identified using pan-lineage markers, and fine subtyping of MAIT cells, M1/M2 macrophages and CD8^+^ T cells subsets was further conducted according to curated subset-specific markers. Marker genes were evaluated based on cluster-specific enriched expression rather than a uniform quantitative threshold, and all annotations were cross-validated by two researchers independently. After cell annotation, only immune cells were retained for downstream analyses. The LASSO gene signature was further evaluated in GSE181483 using ssGSEA. Differences in enrichment scores between healthy and cirrhotic samples were assessed using the Wilcoxon rank-sum test to identify significantly altered immune cell populations.

### 2.7 Functional enrichment analysis

Immune cell populations that were statistically significant across the aforementioned analyses were defined as core immune cells. Based on the median LASSO score, cells were stratified into high-score and low-score groups. Differential expression analysis was performed between the two groups, and genes with adjusted P value < 0.05 and |log2FC| ≥ 0.25 were selected. Gene Ontology (GO) and Kyoto Encyclopedia of Genes and Genomes (KEGG) enrichment analyses were conducted using the clusterProfiler package (28).

### 2.8 Pseudotime analysis

Pseudotime trajectory analysis was performed to characterize dynamic gene expression changes during cellular differentiation. The Monocle package (29) was used to construct developmental trajectories and to investigate the expression dynamics of key genes along the differentiation process. In addition, the TCellSI package (30) was applied to evaluate the functional states of the target cell populations.

### 2.9 Cell-cell communication analysis

Cell-cell communication analysis was conducted to characterize ligand-receptor-mediated signaling interactions among different cell types. The CellChat package (31) was used to systematically model intercellular communication networks based on the integrated ligand-receptor database (CellChatDB.human). Communication probabilities were calculated, interaction networks were constructed, and key signaling pathways were identified. Multidimensional visualization was employed to elucidate coordinated cellular interactions within the cirrhotic microenvironment.

### 2.10 Masson staining

Human liver tissue samples were fixed in 4% paraformaldehyde at room temperature for 24 hours, followed by sequential gradient ethanol dehydration, xylene clearing, and paraffin embedding. Serial sections of 4 μm thickness were cut using a rotary microtome and mounted on glass slides. The sections were deparaffinized in xylene twice (10 minutes each), rehydrated through a graded ethanol series (100%, 95%, 85%, 75%) for 5 minutes each, and finally rinsed with distilled water.

Masson trichrome staining was performed using a commercial kit (Solarbio, Cat. No. G1340) according to the manufacturer’s instructions. Briefly, sections were stained with Weigert’s iron hematoxylin for 5 minutes to label nuclei, rinsed with tap water for 10 minutes, and differentiated with 1% hydrochloric acid alcohol for 3 seconds. After rinsing with distilled water, sections were stained with Ponceau-acid fuchsin solution for 10 minutes, rinsed with distilled water, and differentiated with 1% phosphomolybdic acid solution for 5 minutes. Sections were then directly transferred to aniline blue solution for 5 minutes and differentiated with 1% glacial acetic acid solution for 1 minute. Finally, sections were dehydrated through a graded ethanol series (75%, 85%, 95%, 100%) for 5 minutes each, cleared in xylene twice (10 minutes each), and mounted with neutral balsam.

Images were captured using a bright-field microscope (Olympus, BX53) at 200× magnification, with 5–10 random fields selected per section. The area percentage of collagen fibers (blue staining) was quantitatively analyzed using ImageJ software (National Institutes of Health, USA) to assess the degree of liver fibrosis.

### 2.11 Immunofluorescence staining

Human liver tissue samples from normal, fibrotic, and cirrhotic stages were washed three times with PBS, fixed in 4% paraformaldehyde at room temperature for 15 min, and incubated overnight in 30% sucrose solution at 4 °C for cryoprotection. Tissues were embedded in OCT compound, serially sectioned at 5 μm using a −80 °C cryostat, mounted on poly-L-lysine-coated slides, and air-dried at room temperature.

Tyramide signal amplification (TSA)-based multiplex immunofluorescence staining was performed. Sections were permeabilized with 0.3% Triton X-100 in PBS for 20 min, and endogenous peroxidase was blocked with 3% hydrogen peroxide for 15 min. Non-specific binding sites were blocked with 5% BSA in PBS for 1 h at room temperature. Sections were incubated overnight at 4 °C in a humidified chamber with primary antibodies (1:200 dilution): anti-CD161 (67537-1-Ig), anti-CCL20 (84413-3-RR), anti-CD206 (AFRM0009), anti-CD163 (AF20010), and anti-α-SMA (AF20323). After PBS washing, sections were incubated with HRP-conjugated secondary antibodies, followed by signal amplification with TSA fluorescent dyes (TYR-480, TYR-520, TYR-570, TYR-620, TYR-690). Nuclei were counterstained with DAPI for 5 min in the dark, and sections were mounted with anti-fade mounting medium.

Images were acquired from multiple random fields using a laser scanning confocal microscope. For quantification, 3 patients were included per group, with 2 non-consecutive high-power fields randomly selected per case from lesional areas (fibrotic-cirrhotic interface and adjacent parenchyma), excluding large vessels and overt necrotic regions. Image analysis was performed in ImageJ: nuclei were segmented via Otsu thresholding on the DAPI channel, and cells with fluorescence intensity above the mean background + 2 SD were defined as positive. For colocalization, Manders’ overlap coefficients between CCL20 and each cell marker were calculated using the Coloc 2 plugin, supplemented by the percentage of double-positive cells. Statistical analysis was performed with GraphPad Prism 9.0 using the Mann-Whitney U test; data are presented as mean ± SD, with P < 0.05 considered statistically significant.

### 2.12 Immunohistochemistry staining

Paraffin-embedded human liver tissue sections (4 μm) were deparaffinized in xylene twice (10 minutes each), rehydrated through a graded ethanol series (100%, 95%, 85%, 75%) for 5 minutes each, and rinsed with distilled water. Antigen retrieval was performed by heating sections in citrate buffer (pH 6.0) in a pressure cooker at 121 °C for 15 minutes. After naturally cooling to room temperature, sections were washed three times with PBS (5 minutes each).

Endogenous peroxidase activity was blocked by incubating sections with 3% (v/v) hydrogen peroxide (H_2_O_2_) at room temperature for 10 minutes, followed by three washes with PBS (5 minutes each). Non-specific binding was blocked with 5% BSA in PBS for 1 hour at room temperature. Sections were then incubated with primary antibodies diluted in blocking buffer overnight at 4 °C in a humidified chamber. The primary antibodies used were: anti-Arg1 (1:200, Cat. No. AFM179), anti-AFP (1:200, Cat. No. AFM193), and anti-CCL20 (1:200, Cat. No. 84413-3-RR).

The following day, sections were washed three times with PBS (5 minutes each) and incubated with horseradish peroxidase (HRP)-conjugated secondary antibodies for 30 minutes at room temperature. After washing with PBS, sections were developed with freshly prepared 3,3’-diaminobenzidine (DAB) substrate solution. The color reaction was monitored under a bright-field microscope and terminated by rinsing with distilled water once distinct brown staining was observed (approximately 3–5 minutes). Nuclei were counterstained with hematoxylin for 2 minutes, differentiated with 1% hydrochloric acid-ethanol solution for 3 seconds, and blued in running tap water for 10 minutes. Sections were then dehydrated through a graded ethanol series, cleared in xylene, and mounted with neutral balsam.

Bright-field images were captured using an Olympus BX53 microscope at 200× magnification to compare the expression localization and intensity of target proteins between normal and cirrhotic liver tissues.

### 2.13 Virtual knockout

In silico virtual knockout analysis was performed using the scTenifoldKnk R package (32) to characterize the regulatory function of the identified core gene in the target immune cell subset.

The target cell population was first isolated from cirrhotic samples of the single-cell transcriptomic dataset, and its raw count expression matrix was extracted via the Seurat package; the top 3,000 highly variable genes screened by the variance stabilizing transformation method were then combined with the target gene to construct the input expression matrix. The analytical workflow comprised construction of a wild-type single-cell gene regulatory network, generation of a pseudo-knockout network via in silico ablation of the target gene, and manifold alignment between the two networks, with Z-scores and adjusted P calculated for each gene to quantify the transcriptional perturbation induced by target gene knockout. Key analytical parameters included a mitochondrial read ratio threshold of 0.1, a minimum library size of 500 per cell, and 10 sub-networks with 500 randomly sampled cells per sub-network.

Genes with an adjusted P < 0.05 were defined as significantly differentially regulated genes, and the top 20 genes ranked by descending Z-score were subjected to Gene Ontology functional module enrichment analysis, with the Benjamini–Hochberg procedure applied for multiple testing correction.

### 2.14 Statistical analysis

All statistical analyses were performed using R software (version 4.4.1). For all differential expression analyses, gene set enrichment analyses, module–phenotype correlation tests, immune cell infiltration comparisons, cell functional state assessments, and correlation analyses, the Benjamini–Hochberg (BH) procedure was uniformly applied for multiple-testing correction to control the false discovery rate (FDR). Adjusted P were reported, and statistical significance was defined as adjusted P < 0.05 unless otherwise specified.

For LASSO regression, feature selection was based on coefficient shrinkage via 10-fold cross-validation, and no additional multiple-testing correction was applied. For cell–cell communication analysis, statistical significance of ligand–receptor interactions was determined by the built-in permutation test of the CellChat package.

Notably, for single-cell transcriptomic analyses involving large cell numbers, extremely small P may arise from elevated statistical power; all relevant conclusions are interpreted in conjunction with effect size magnitudes and biological relevance, rather than relying exclusively on significance thresholds.

### 2.15 Ethics statement

The studies involving human participants and human liver tissue samples were reviewed and approved by the Ethics Committee of The Second Affiliated Hospital of Zhejiang Chinese Medical University (Approval No. 2024-053-01; Date of approval: July 17, 2024). All patients/participants provided written informed consent to participate in this study and for the use of their tissue samples. The study was conducted in accordance with the Declaration of Helsinki and Good Clinical Practice (GCP) guidelines.

## 3 Results

### 3.1 Identification of phenotype-associated gene modules

To identify differentially expressed genes (DEGs) between cirrhosis and mild liver fibrosis, gene expression data from 40 mild fibrosis samples and 32 cirrhosis samples in Dataset 1 were first normalized (**Supplementary Material 1**, **Supplementary Figure 1A**). Using thresholds of adjusted P < 0.05 and |log2 fold change (FC)| ≥ 0.25, a total of 642 DEGs were identified, comprising 379 upregulated and 263 downregulated genes (**Supplementary Material 2**, **Supplementary Table 1**).

**Figure 1:**
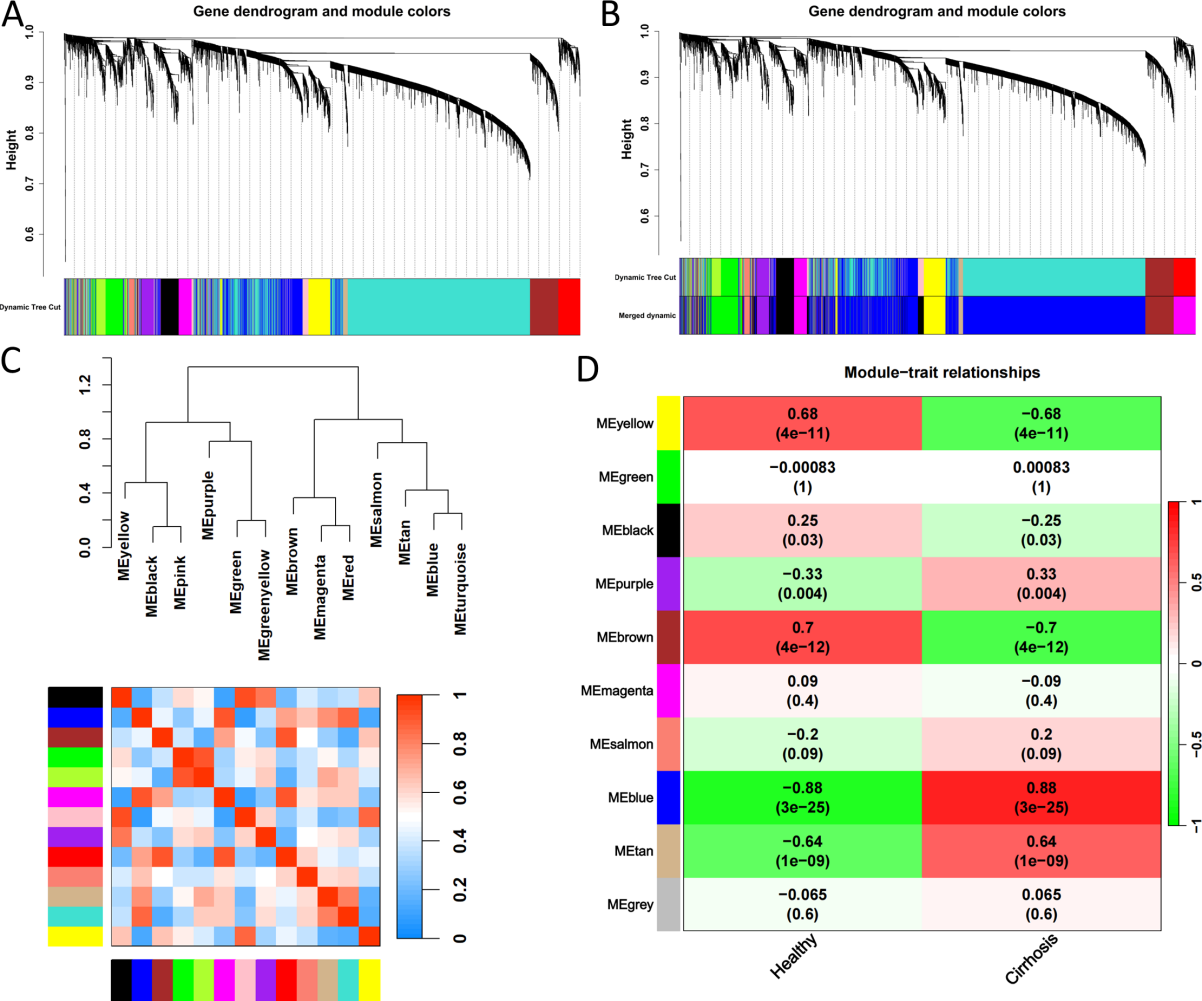
Gene clustering dendrograms and module-phenotype correlations in WGCNA for liver cirrhosis. **(A)** Gene clustering dendrogram and module colors identified by the dynamic tree cut method; **(B)** Gene clustering dendrogram and module colors following module merging; **(C)** Dendrogram of gene module correlations and the corresponding correlation heatmap; **(D)** Heatmap showing the correlation between gene modules (MEblue, MEbrown, etc.) and clinical phenotypes (healthy vs cirrhosis).

To further delineate gene modules associated with the cirrhotic phenotype, weighted gene co-expression network analysis (WGCNA) was performed on combined normal and cirrhotic samples from Dataset 2. The optimal soft-thresholding power was determined (**Supplementary Material 1**, **Supplementary Figures 1B–D**), and dynamic tree cutting identified 13 initial co-expression modules (**Figure 1A**). To enhance module specificity and reduce redundancy, modules exhibiting an inter-module correlation coefficient greater than 0.75 were merged (**Figure 1C**), ultimately yielding 10 distinct gene co-expression modules (**Figure 1B**). Correlation analysis between module eigengenes (MEs) and the cirrhosis phenotype revealed that the MEblue and MEbrown modules were most strongly associated with cirrhosis (|r| ≥ 0.7, adjusted P < 0.05) (**Figure 1D**; **Supplementary Material 2**, **Supplementary Table 2**), suggesting that these modules may mediate key biological functions in the progression of liver cirrhosis.

### 3.2 Identification of core gene sets via machine learning

To identify core immune-related genes, a comprehensive strategy integrating differential expression analysis, weighted gene co-expression network analysis (WGCNA) module-trait associations, and immune gene set enrichment was employed to screen candidate genes strongly associated with disease status for subsequent machine learning analysis.

**Figure 2:**
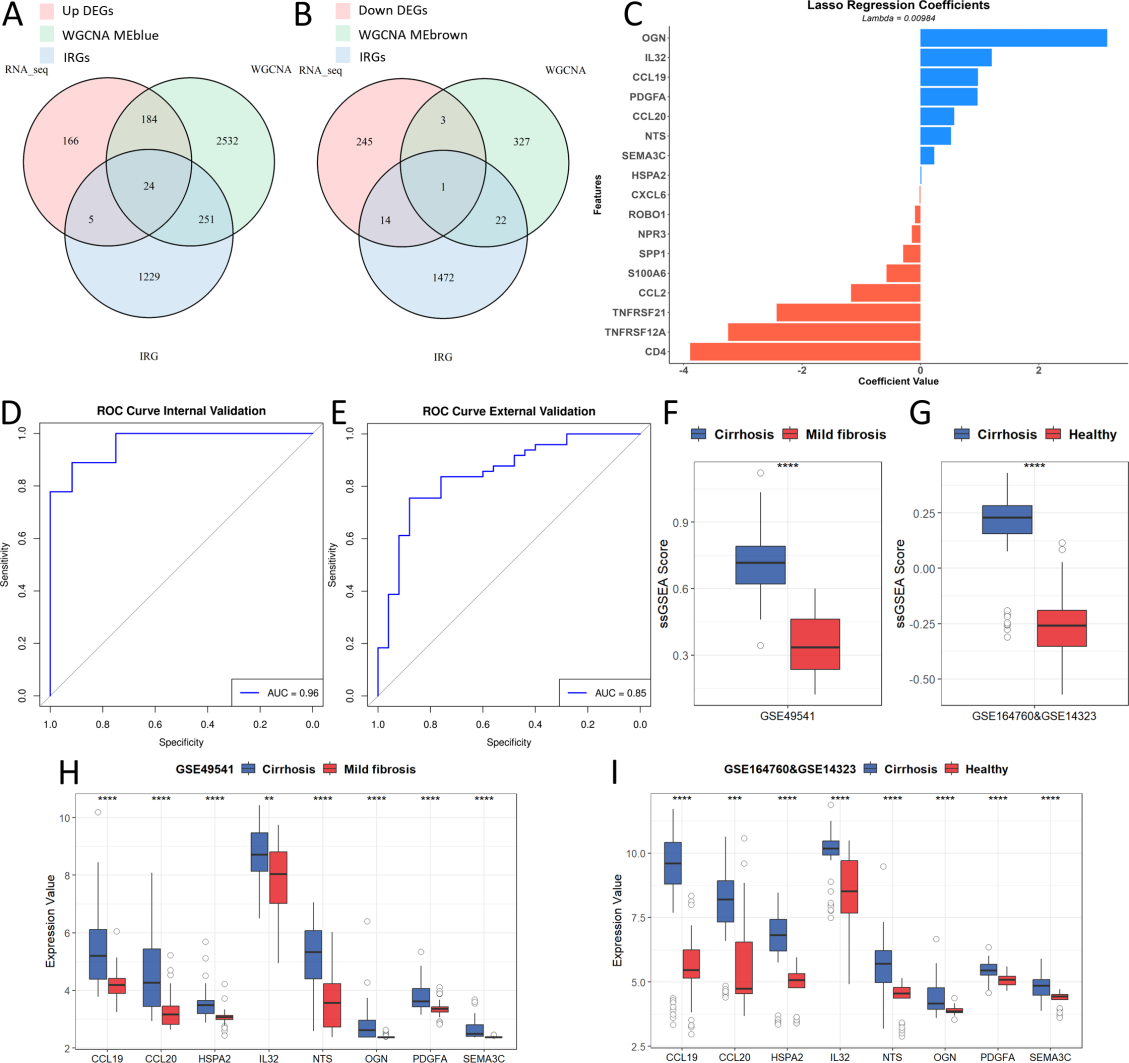
Screening and validation of core immune-related genes in liver cirrhosis via integrative analysis. **(A)** Venn diagram showing the intersection of upregulated DEGs, MEblue module genes, and IRGs (24 candidate genes); **(B)** Venn diagram showing the intersection of downregulated DEGs, MEbrown module genes, and IRGs (1 candidate gene); **(C)** LASSO regression coefficient profiles identifying 17 core genes with non-zero coefficients; **(D)** ROC curve for internal validation (AUC = 0.96); **(E)** ROC curve for external validation (AUC = 0.85); **(F)** Boxplots comparing ssGSEA scores of the LASSO gene set between cirrhosis and mild fibrosis groups in Dataset 1; **(G)** Boxplots comparing ssGSEA scores of the LASSO gene set between cirrhosis and healthy groups in Dataset 2; **(H, I)** Boxplots comparing expression levels of the 8 core genes (*OGN*, *IL32*, *CCL19*, *PDGFA*, *CCL20*, *NTS*, *SEMA3C*, *HSPA2*) between cirrhosis and control groups in Dataset 1 and Dataset 2. Statistical significance: ns, not significant (P > 0.05); *P < 0.05; **P < 0.01; ***P < 0.001; ****P < 0.0001.

The intersection of significantly upregulated differentially expressed genes (DEGs) from Dataset 1, the MEblue module (r ≥ 0.7), and immune-related genes obtained from the ImmPort database yielded 24 candidate genes (**Figure 2A**; **Supplementary Material 2**, **Supplementary Table 3**). Similarly, intersecting significantly downregulated DEGs, the MEbrown module (r ≤ −0.7), and the immune-related gene set identified one candidate gene (**Figure 2B**; **Supplementary Material 2**, **Supplementary Table 3**).

Feature selection was then performed on the resulting 25 genes using least absolute shrinkage and selection operator (LASSO) regression with 10-fold cross-validation (**Supplementary Material 1**, **Supplementary Figures 2A, B**), leading to the identification of 17 core genes with robust classification performance (**Figure 2C**). The model demonstrated excellent discriminative ability in the training set (AUC = 0.96; **Figure 2D**) and maintained strong performance in an independent external validation set (AUC = 0.85; **Figure 2E**), indicating good generalizability and potential clinical utility.

A LASSO-derived gene signature comprising eight genes with positive coefficients (*OGN, IL32, CCL19, PDGFA, CCL20, NTS, SEMA3C, HSPA2*) was subsequently constructed. Single-sample gene set enrichment analysis (ssGSEA) performed in both Dataset 1 and Dataset 2 showed significantly higher immune activity scores in the cirrhosis group than in the mild fibrosis group (Dataset 1) and the healthy group (Dataset 2), respectively (adjusted P < 0.0001 for all comparisons; **Figures 2F, G**). Furthermore, rank-sum tests evaluating the expression levels of these eight genes in both datasets demonstrated significantly higher expression in the cirrhosis group compared with controls (adjusted P < 0.01 for *IL32* in Dataset 1; adjusted P < 0.0001 for the remaining genes across both datasets; **Figures 2H, I**). These results not only support the biological relevance of the gene signature but also strengthen its potential utility as a biomarker of immune dysregulation in cirrhosis.

### 3.3 Multiple immune infiltration analyses reveal features of the cirrhosis immune microenvironment

**Figure 3:**
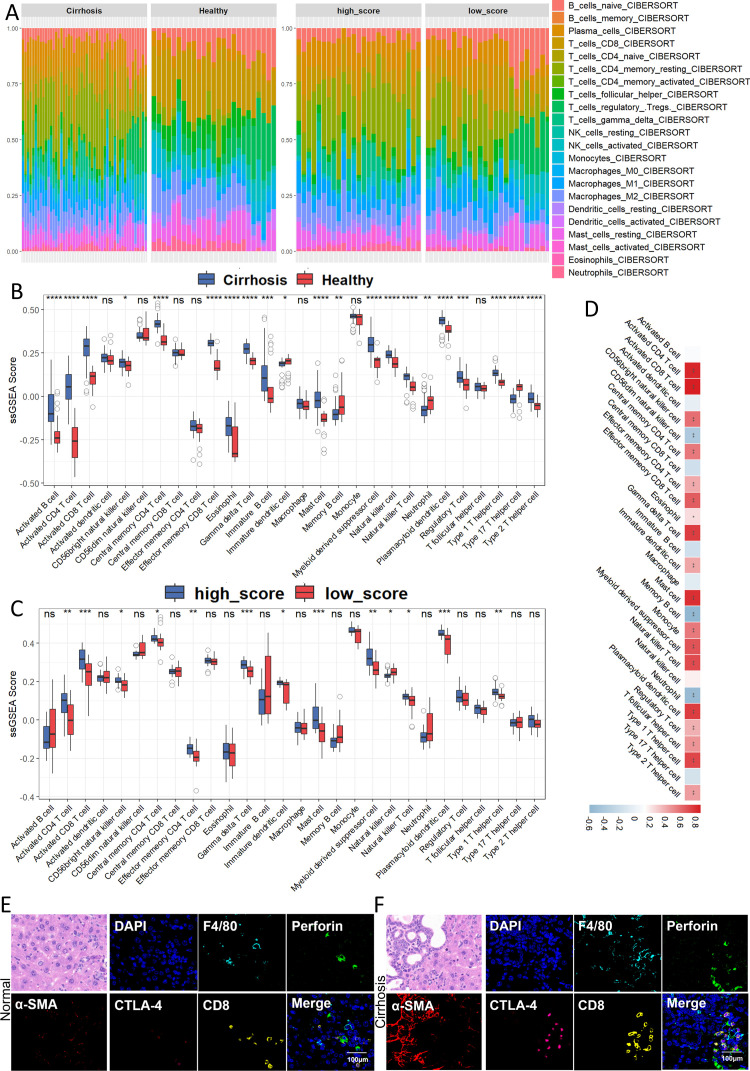
Immune infiltration characteristics of the immune microenvironment in liver cirrhosis. **(A)** Heatmap of infiltrating immune cell proportions in healthy, cirrhotic, high-score, and low-score samples; **(B)** Boxplots comparing ssGSEA enrichment scores of immune cell signatures between healthy and cirrhotic groups (Wilcoxon rank-sum test); **(C)** Boxplots comparing ssGSEA enrichment scores between high-score and low-score subgroups (Wilcoxon rank-sum test); **(D)** Spearman correlation heatmap between LASSO signature ssGSEA scores and 28 immune cell signature scores; **(E, F)** Immunofluorescence staining of normal and cirrhotic liver tissues, showing expression of F4/80 (macrophage marker), α-SMA (activated hepatic stellate cell marker), CTLA-4 (a co-inhibitory receptor involved in T-cell regulation and dysfunction), CD8, and Perforin (cytotoxic effector molecule of T cells). Statistical significance: ns, not significant (P > 0.05); *P < 0.05;**P < 0.01; ***P < 0.001; ****P < 0.0001.

To systematically characterize the immune microenvironment during the progression of cirrhosis, the CIBERSORT algorithm implemented in the IOBR package was applied to Dataset 2 to estimate immune cell infiltration profiles in Cirrhosis and Healthy samples. Based on the key gene set identified by LASSO regression, single-sample gene set enrichment analysis (ssGSEA) was subsequently performed to calculate enrichment scores for each sample. Using the median score as the threshold, cirrhosis samples in Dataset 2 were further stratified into high_score and low_score subgroups. CIBERSORT was then re-applied to compare immune infiltration patterns between these two subgroups, generating immune cell infiltration profiles for Cirrhosis versus Healthy samples as well as for the high_score and low_score groups (**Figure 3A**). This approach aimed to reveal heterogeneity in the immune landscape among cirrhosis patients with distinct molecular phenotypes.

To identify immune cell types significantly associated with disease status, we integrated 28 immune cell-specific gene signature sets defined in the TISIDB database and performed a comprehensive ssGSEA analysis on Dataset 2. Wilcoxon rank-sum tests were used to compare enrichment scores between the Healthy and Cirrhosis groups and between the high_score and low_score subgroups. In the comparison between Healthy and Cirrhosis samples, The ssGSEA scores for Activated CD4^+^ T cells, Gamma delta T cells, Myeloid-derived suppressor cells, Type 1 T helper cells, Natural killer T cells, Central memory CD4^+^ T cells, Effector memory CD8^+^ T cells, Activated CD8^+^ T cells, Plasmacytoid dendritic cells, Type 2 T helper cells, Mast cells, and Activated B cells showed strong statistical significance (adjusted P < 0.0001) and were significantly enriched in the Cirrhosis group, indicating elevated transcriptional activity of these immune cell signatures (**Figure 3B**). In the comparison between the high_score and low_score subgroups, Activated CD8^+^ T cells, Gamma delta T cells, Plasmacytoid dendritic cells, Mast cells, and Activated CD4^+^ T cells were significantly enriched (adjusted P < 0.001) (**Figure 3C**).

To further explore the biological significance of the LASSO-selected gene set and its relationship with immune microenvironment remodeling, we assessed correlations between the ssGSEA score of the LASSO gene set and the ssGSEA scores of the 28 immune cells signatures. Spearman correlation analysis showed that Activated CD4^+^ T cells, Activated CD8^+^ T cells, Effector memory CD8^+^ T cells, Gamma delta T cells, Natural killer T cells, Mast cells, Myeloid-derived suppressor cells, Plasmacytoid dendritic cells, and Type 1 T helper cells were significantly and positively correlated with the LASSO gene set score (adjusted P < 0.01) (**Figure 3D**). Immunofluorescence staining further supported these findings, demonstrating distinct T-cell activation states in cirrhosis. Specifically, increased macrophage infiltration (F4/80), enhanced activation of CD8^+^ T cells indicated by CTLA-4 upregulation, and a modest increase in perforin expression were observed, suggesting phenotypic heterogeneity and differential activation states of T cells in cirrhotic livers (**Figures 3E, F**). Quantitative analysis of immunofluorescence staining (**Supplementary Material 1**, **Supplementary Figure 2C**) confirmed these observations: cirrhotic livers showed significantly increased F4/80^+^ macrophages (P < 0.01), CTLA-4^+^ cells (P < 0.001) and CD8^+^ T cells (P < 0.01). These quantitative experimental results directly support our conclusion.

Collectively, these results indicate that cirrhosis progression is closely associated with immune microenvironment remodeling, particularly with elevated transcriptional signatures of CD8^+^ T cell activation. The transcriptional activity of multiple immune cell gene sets, including activated CD4^+^/CD8^+^ T cells and Th1 cells, was significantly increased in the Cirrhosis group, suggesting widespread transcriptional activation of both adaptive and innate immune programs. Moreover, the high_score subgroup showed significant enrichment of several immune cell signatures (e.g., Activated CD8^+^ T cell, Gamma delta T cell, Plasmacytoid dendritic cell), and the ssGSEA score of the LASSO gene set was strongly correlated with multiple immune cell signatures. These multi-dimensional immune infiltration analyses collectively suggest that CD8^+^ T cells play a critical role in the progression of cirrhosis.

### 3.4 Validation of the central role of CD8^+^ T cells in cirrhosis using single-cell transcriptomic analysis

**Figure 4:**
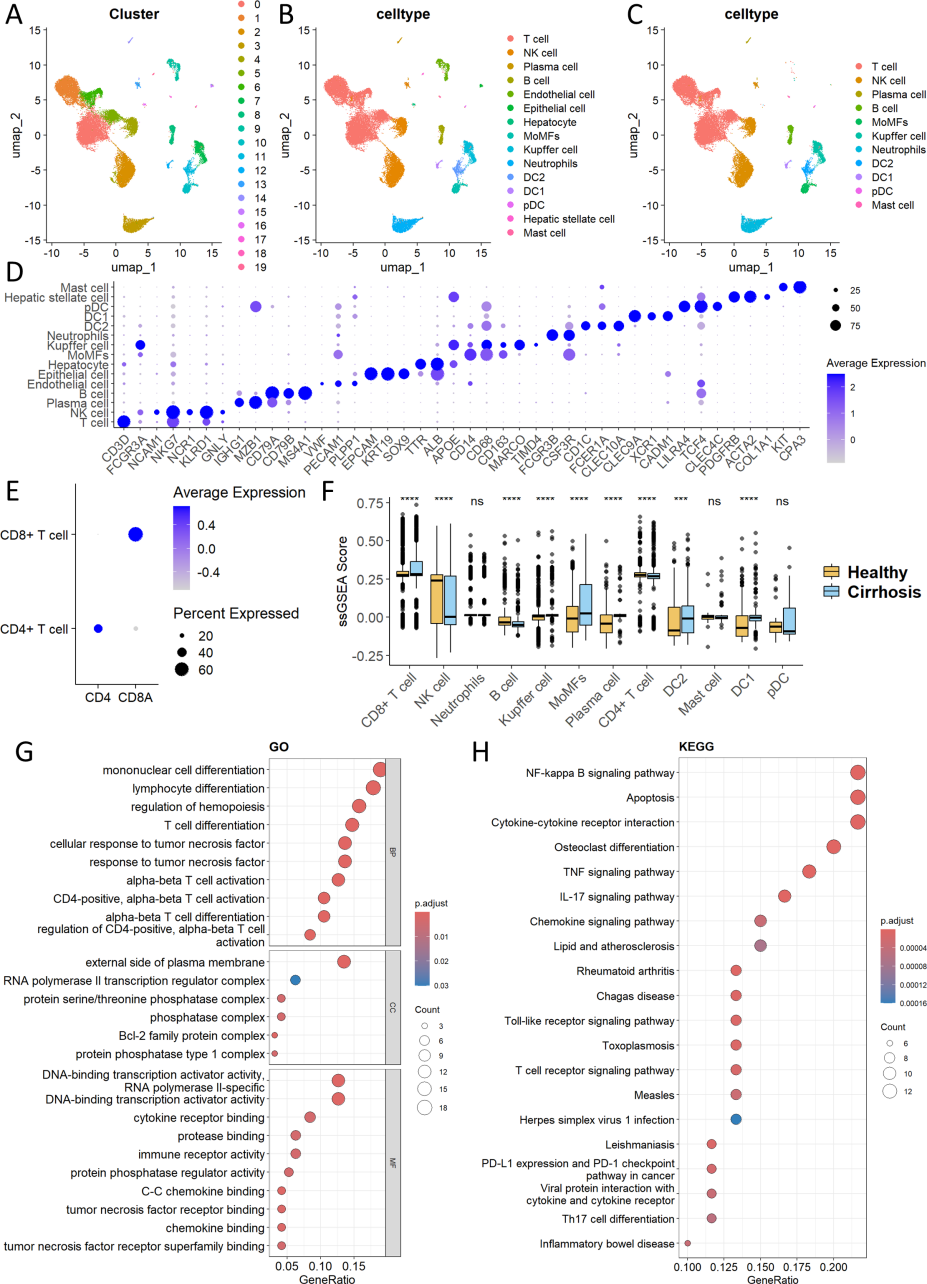
Single-cell transcriptomic validation of CD8^+^ T cells as core immune cells in liver cirrhosis. **(A)** UMAP plot of 20 distinct cell clusters in cirrhotic liver tissue; **(B)** UMAP plot with annotations of major cell populations; **(C)** UMAP plot highlighting immune cell populations; **(D)** Bubble plot showing expression of canonical marker genes across immune cell types; **(E)** Bubble plot displaying marker genes distinguishing CD4^+^ and CD8^+^ T cell subsets; **(F)** Boxplots comparing ssGSEA enrichment scores of immune cell signatures between healthy and cirrhotic groups (Wilcoxon rank-sum test). **(G)** Bubble plot illustrating Gene Ontology (GO) enrichment analysis results for differentially expressed genes identified in the CD8^+^ T cell high-score group. **(H)** Bubble plot illustrating Kyoto Encyclopedia of Genes and Genomes (KEGG) pathway enrichment analysis results for differentially expressed genes identified in the CD8^+^ T cell high-score group. Statistical significance: ns, not significant (P > 0.05); ***P < 0.001; ****P < 0.0001.

To further elucidate the role of CD8^+^ T cells in the progression of cirrhosis and to explore the underlying molecular mechanisms, single-cell RNA sequencing (scRNA-seq) data from the GSE181483 dataset were analyzed. Unsupervised clustering identified 20 distinct cell clusters (**Figure 4A**). Following cell-type annotation, immune cell populations were retained for downstream analysis (**Figures 4B–D**).

To validate the pivotal role of the immune system in cirrhosis progression and to define key functional immune subsets, T cells were first classified into CD4^+^ and CD8^+^ subpopulations (**Figure 4E**). A LASSO-derived robust gene signature was then applied to single-sample gene set enrichment analysis (ssGSEA) to quantify the activity levels of individual immune cell types. Nonparametric rank-sum testing revealed significantly higher ssGSEA scores of the LASSO signature in CD8^+^ T cells, monocyte-derived macrophages (MoMFs), and type 1 dendritic cells (DC1) from cirrhotic tissues compared with healthy controls (adjusted P < 0.0001; **Figure 4F**). These findings indicate enhanced transcriptional activity of the cirrhosis-associated immune gene program in these subsets, which correlates with their potential involvement in local inflammatory responses and immune regulation.

Notably, CD8^+^ T cells consistently exhibited the most pronounced upregulation across both bulk immune infiltration analyses and single-cell transcriptomic profiling, identifying them as a central immune subset across multiple analytical platforms. Given their established roles in immune surveillance, cytotoxic effector function, and chronic inflammation, we hypothesized that functional heterogeneity within CD8^+^ T cells may critically influence disease progression.

Accordingly, cirrhotic samples were stratified into high- and low-CD8^+^ T-cell activity groups using the median ssGSEA score as the cutoff. Differential expression analysis between these groups identified significantly differentially expressed genes (DEGs) (adjusted P < 0.05 and logFC ≥ 0.25). Gene Ontology (GO) and Kyoto Encyclopedia of Genes and Genomes (KEGG) enrichment analyses were performed using the ClusterProfiler package, with adjusted P < 0.05 set as the significance threshold.GO enrichment analysis demonstrated that the DEGs were primarily involved in immune-related biological processes, including “mononuclear cell differentiation” “lymphocyte differentiation” “regulation of hemopoiesis” “cellular response to tumor necrosis factor” and “response to tumor necrosis factor”(**Figure 4G**). These results indicate that elevated CD8^+^ T-cell activity is not an isolated phenomenon but is associated with a broader network of immune activation and remodeling. KEGG pathway analysis further highlighted enrichment in the “NF-κB signaling pathway” “apoptosis” “cytokine-cytokine receptor interaction” “TNF signaling pathway” and “IL-17 signaling pathway” (**Figure 4H**). These pathways represent critical nodes linking innate and adaptive immunity and have been widely implicated in persistent inflammation, hepatocyte injury, and extracellular matrix remodeling during the transition from liver fibrosis to cirrhosis (33–36).

### 3.5 Pseudotime analysis identifies *CCL20* as a key immune gene and MAIT cells as a target subpopulation

**Figure 5:**
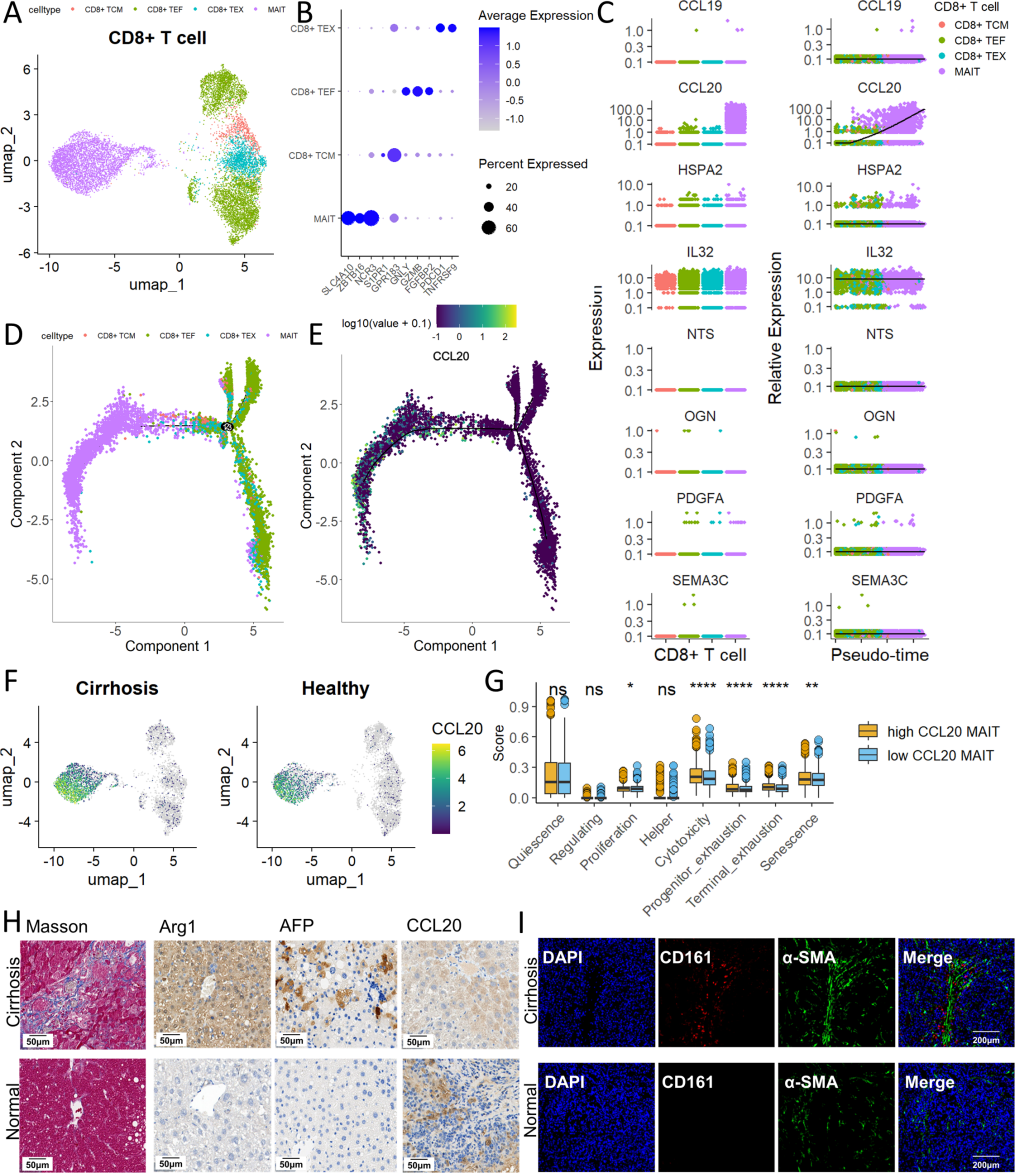
Pseudotime analysis identifies *CCL20* as a key gene regulating MAIT cells function in liver cirrhosis. **(A)** UMAP plot showing clustering of CD8^+^ T cell subsets (CD8^+^ TCM, CD8^+^ TEF, CD8^+^ TEX, MAIT cells); **(B)** Bubble plot of canonical marker genes across CD8^+^ T cell subsets; **(C)** Scatter plot with fitted line illustrating expression dynamics of *CCL20* and other key genes along the pseudotime trajectory; **(D)** Pseudotime trajectory plot of CD8^+^ T cell differentiation paths; **(E)** UMAP feature plot highlighting *CCL20* expression across CD8^+^ T cells; **(F)** UMAP plots comparing *CCL20* expression in CD8^+^ T cells from healthy controls and cirrhotic patients; **(G)** Boxplots comparing T cell functional scores between *CCL20*-high and *CCL20*-low MAIT cells subsets.; **(H)** Masson staining, arginase 1 (Arg1), alpha-fetoprotein (AFP), and CCL20 immunohistochemical staining of normal and cirrhotic liver tissues; **(I)** Immunofluorescence staining of CD161 (MAIT cells marker), α-SMA, and DAPI in normal and cirrhotic liver tissues. Statistical significance: ns, not significant (P > 0.05); *P < 0.05; **P < 0.01; ****P < 0.0001.

To investigate the dynamic differentiation trajectories of CD8^+^ T cells during cirrhosis progression and identify key regulators of their functional states, single-cell subset annotation was performed on CD8^+^ T cell populations, followed by pseudotime analysis to reconstruct temporal differentiation trajectories. This analysis revealed four major CD8^+^ T cell lineages: central memory CD8^+^ T cells (CD8^+^ TCM), effector CD8^+^ T cells (CD8^+^ TEF), exhausted CD8^+^ T cells (CD8^+^ TEX), and MAIT cells (**Figures 5A, B**).

*CCL20* was specifically expressed in MAIT cells, with expression levels dynamically varying along the pseudotime trajectory (**Figure 5C**) and predominantly localized within MAIT cells (**Figures 5D, E**). Notably, *CCL20* expression in MAIT cells was significantly higher in cirrhosis patients compared with healthy controls, suggesting a potential role in immune cell recruitment or functional modulation within the pathological microenvironment (**Figure 5F**). To further examine the relationship between *CCL20* expression and MAIT cells functional states, MAIT cells from cirrhosis patients were stratified into low- and high-*CCL20* expression groups based on the median *CCL20* expression. Functional state scoring using the TcellSI system (based on transcriptional signatures) and subsequent rank-sum tests demonstrated that high-CCL20 MAIT cells had significantly higher scores for cytotoxicity, progenitor exhaustion, and terminal exhaustion gene signatures (adjusted P < 0.0001), with the cytotoxicity signature showing the most pronounced elevation (**Figure 5G**). These results indicate that CCL20 expression is associated with transcriptional profiles indicative of enhanced cytotoxic potential and exhaustion progression in MAIT cells, and may contribute to MAIT cell recruitment and functional state transition.

To confirm the pathological identity of the human liver specimens used in this study, we next performed histopathological validation. Masson trichrome staining verified prominent collagen deposition and fibrotic septum formation in cirrhotic tissues; Arg1 immunohistochemistry confirmed the accumulation of M2-polarized macrophages, the key interacting partner of MAIT cells in our proposed mechanism; and AFP immunohistochemistry was included as an auxiliary marker of hepatocyte dedifferentiation in advanced cirrhosis. Alongside these validations, CCL20 immunohistochemistry demonstrated markedly increased CCL20 protein levels in cirrhotic liver tissues (**Figure 5H**). This finding was further corroborated by immunofluorescence staining, which confirmed elevated CCL20 expression alongside the MAIT cell marker CD161 in fibrotic areas of cirrhotic liver tissue (**Figure 5I**).

### 3.6 Cell-cell communication analysis reveals a pathology-specific CCL20-CCR6 axis associated with MAIT-M2 macrophage interactions and positive feedback regulation in liver cirrhosis

To investigate the dynamic intercellular communication networks within the cirrhotic immune microenvironment and elucidate the roles of key cell subsets in disease progression, we performed systematic cell-cell communication analysis on the cirrhotic samples from dataset GSE181483 using the CellChat tool. Global cell-cell interaction profiling revealed extensive crosstalk between MAIT cells and multiple immune cell subsets, with particularly robust interactions notably with MoMFs, CD8^+^ TCM, NK cells, and CD8^+^ TEX cells (**Supplementary Material 1**, **Supplementary Figures 3A–D**). Quantitative assessment of ligand-receptor interaction strength using bubble plots (**Supplementary Material 1**, **Supplementary Figures 3E, F**) revealed that despite the broad interactome of MAIT cells, only the CCL20-CCR6 signaling axis exhibited statistically significant and specific signaling activity between MAIT cells and MoMFs. Based on these findings, we directed our subsequent mechanistic studies toward the functional crosstalk between MAIT cells and MoMFs mediated by the CCL20-CCR6 axis.

**Figure 6:**
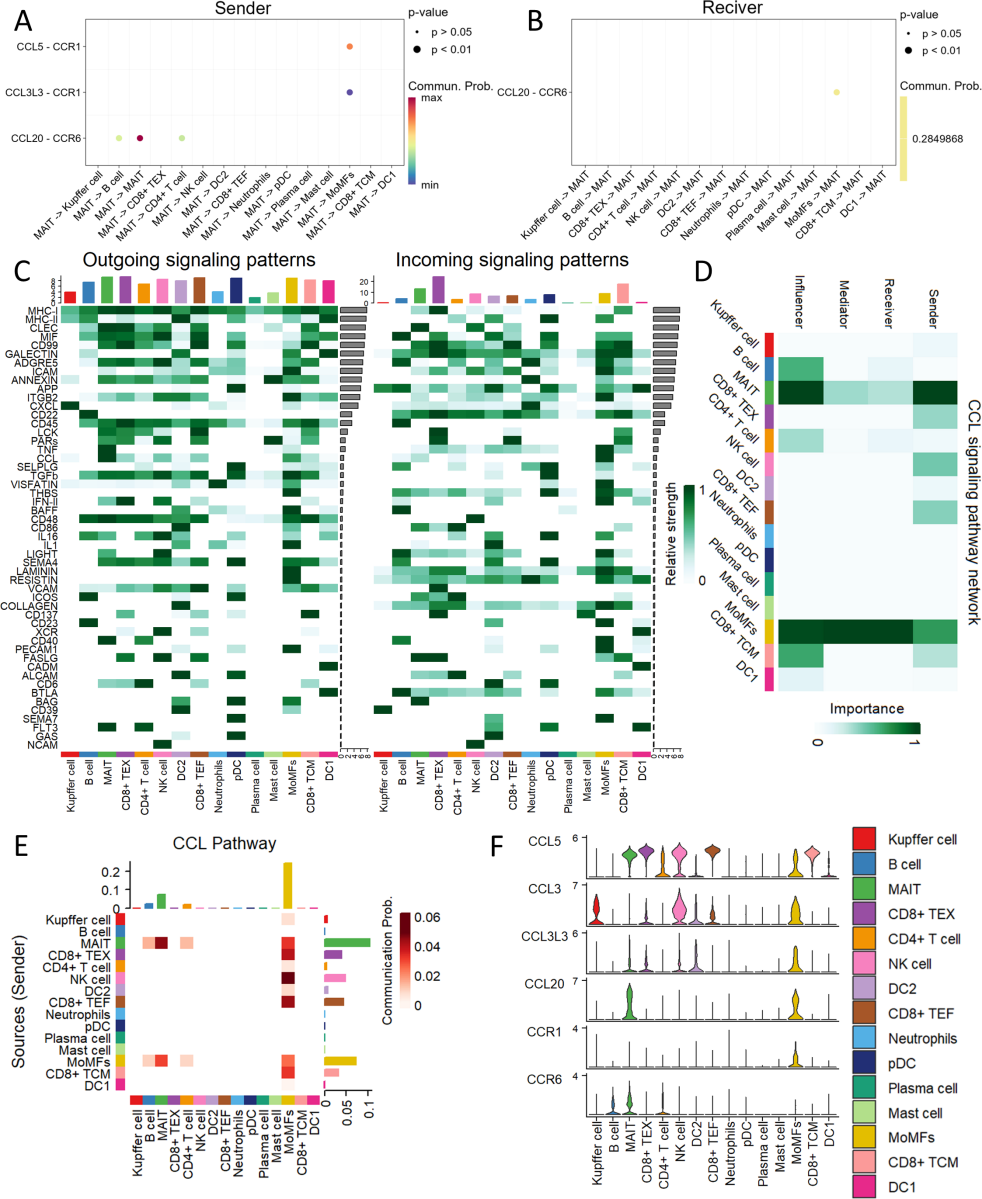
Cell-cell communication analysis reveals an association between the CCL20-CCR6 axis and MAIT-MoMFs crosstalk in liver cirrhosis. **(A, B)** Bubble plots showing communication probabilities of ligand-receptor pairs associated with the CCL signaling pathway in MAIT cells; **(C)** Heatmap illustrating outgoing and incoming signaling roles of each cell type in the CCL pathway; **(D)** Heatmap depicting relative importance of cellular roles in the CCL signaling network; **(E)** Heatmap of intercellular communication probabilities within the CCL pathway; **(F)** Violin plots showing expression distribution of CCL family genes across immune cell types.

Further examination using bubble plots indicated that MAIT cells can engage in autocrine regulation via the secretion of CCL20 (**Figure 6A**). Concurrently, MoMFs were found to participate in the paracrine regulation of MAIT cells by expressing CCL20 (**Figure 6B**), suggesting the existence of a potential positive feedback loop within this inflammatory chemotactic signaling pathway. At the pathway level, analysis of the CCL chemokine signaling network showed that MAIT cells receive strong incoming signals from MoMFs while simultaneously acting as major contributors of outgoing signals through this pathway (**Figures 6C, D**). Heatmap visualization further clarified that the CCL pathway activity in MAIT cells is primarily driven by autocrine signaling (**Figure 6E**), whereas MoMFs predominantly utilize this pathway to exert regulatory effects on MAIT cells, highlighting their immunomodulatory potential.

Strikingly, *CCL20* gene expression was found to be specifically and almost exclusively distributed within the MAIT and MoMFs populations, with minimal expression detected in other cell types (**Figure 6F**). This highly specific expression pattern suggests that the CCL20-CCR6 ligand-receptor axis constitutes a privileged communication node within the cirrhotic microenvironment. Collectively, these findings reveal that CCL20 is associated with a specific and potent bidirectional immune interaction between MAIT cells and MoMFs, and may contribute to a positive feedback regulatory mechanism that sustains chronic inflammation and promotes further immune cell recruitment in liver cirrhosis.

To further elucidate the specific interaction network between MAIT cells and MoMFs within the cirrhotic microenvironment and to identify key molecular mechanisms underlying chronic inflammation and immune regulation, MoMFs were functionally annotated and subdivided into pro-inflammatory M1 and anti-inflammatory/reparative M2 subsets (**Supplementary Material 1**, **Supplementary Figure 3C**). Comprehensive cell-cell communication analyses were subsequently performed in conjunction with MAIT cells.

**Figure 7:**
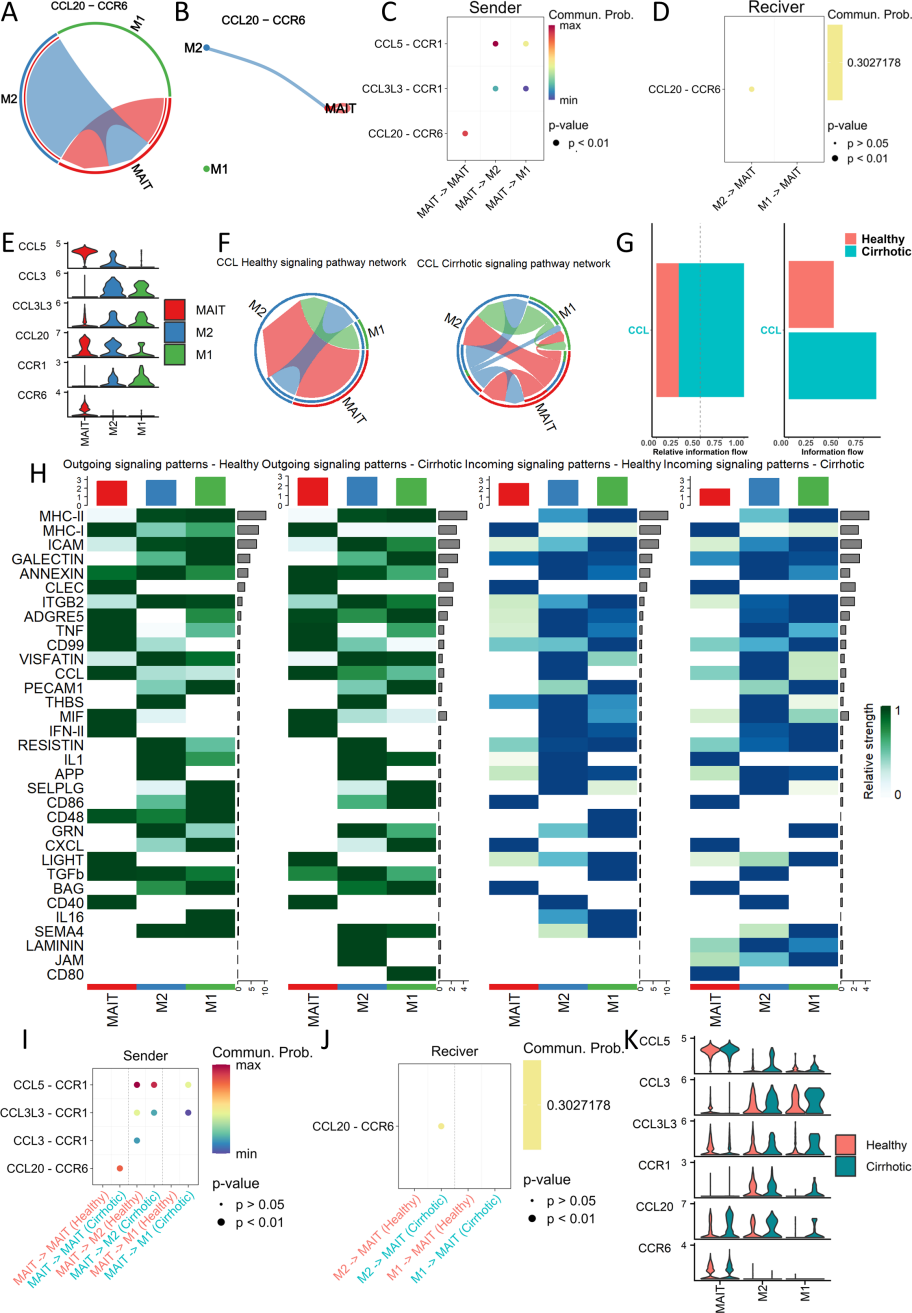
The CCL20-CCR6 axis is associated with cirrhosis-specific crosstalk between MAIT cells and M2 macrophages. **(A)** Circular interaction network illustrating communication among M1, M2, and MAIT cells along the CCL20-CCR6 axis; **(B)** Schematic representation of intercellular communication among M1, M2, and MAIT cells within the CCL20-CCR6 axis. **(C, D)** Bubble plots showing communication probabilities of ligand-receptor pairs associated with the CCL signaling pathway. **(E)** Violin plots depicting the expression distribution of CCL family genes in MAIT, M2, and M1 cells. **(F)** Circular network of the CCL signaling pathway in Healthy and Cirrhosis groups. **(G)** Comparison of overall CCL pathway signaling strength between Healthy and Cirrhosis groups. **(H)** Heatmaps illustrating outgoing and incoming CCL pathway signaling across cell types in Healthy and Cirrhosis groups. **(I, J)** Bubble plots of communication probabilities for CCL pathway-associated ligand-receptor pairs under Healthy and Cirrhosis conditions. **(K)** Violin plots showing the expression distribution of CCL family genes in Healthy and Cirrhosis groups.

In-depth analysis of the Cirrhosis group from the GSE181483 dataset demonstrated no significant ligand-receptor interactions between M1 and MAIT cells along the CCL20-CCR6 signaling axis. In contrast, M2 and MAIT cells exhibited strong bidirectional communication. Specifically, M2 cells highly expressed CCL20 and mediated paracrine regulation of MAIT cells, whereas CCL20 expression in M1 cells was markedly lower than that in M2 and MAIT cells (**Figures 7A–E**). These findings suggest that M2 macrophages may exert non-classical immunomodulatory functions during cirrhosis progression by promoting MAIT cell recruitment and local accumulation.

To evaluate the pathological specificity of this pathway, cellular communication networks were compared between healthy controls (Healthy) and cirrhotic samples (Cirrhosis). In healthy liver tissue, no significant CCL pathway interactions were observed between M2 and MAIT cells, and MAIT cells did not display a prominent autocrine regulatory pattern. By contrast, in cirrhotic conditions, the M2-to-MAIT regulatory axis was activated, accompanied by the specific emergence of a MAIT-intrinsic CCL autocrine loop (**Figure 7F**). Moreover, global analysis of the CCL signaling pathway revealed significantly higher overall signaling strength in the cirrhosis group than in the healthy group (**Figure 7G**). Heatmap visualization further confirmed that both M2 cells (as major ligand sources) and MAIT cells (as principal signal recipients) exhibited significantly increased contributions and signal reception within the CCL pathway under disease conditions (**Figure 7H**).

Refined analysis using bubble plots, with a focus on the CCL20-CCR6 axis, demonstrated that M2-mediated regulation of MAIT cells via CCL20 occurred exclusively in cirrhosis. Concurrently, MAIT cells engaged in autocrine signaling through the CCL20-CCR6 axis (**Figures 7I, J**). These results indicate that the functional M2-to-MAIT interaction and the MAIT-specific CCL20-CCR6 self-regulatory circuit are features of the pathological state and are largely absent under physiological conditions. Consistently, violin plot analysis showed that CCL20 expression was highly enriched in M2 and MAIT cells in the Cirrhosis group and was significantly elevated compared with the Healthy group (**Figure 7K**).

To characterize the regulatory function of *CCL20* in MAIT cells during cirrhosis, we performed in silico virtual knockout analysis using the scTenifoldKnk algorithm.

We identified 16 significantly differentially regulated genes (adjusted P < 0.05), all upregulated after *CCL20* ablation (**Supplementary Material 1**, **Supplementary Figures 4A, B**). These genes fall into four functional categories: chemokine ligands (*CCL4L2, CCL4, CCL5, CCL3L3*), T cell activation markers (*CD160, CD69, TRGV3, TRGC2*), inflammatory signaling mediators (*IFNG, NFKBIA*), and cellular stress/survival regulators (*BCL2A1, GPR65, HSPA1A, HSPA1B, TXNIP*).

Functional module enrichment revealed that *CCL20* knockout perturbed multiple core biological programs of MAIT cells, dominated by chemokine/inflammatory recruitment, followed by TCR/NK-like activation, NF-κB signaling, tissue retention, stress response, and IFN effector function (**Supplementary Material 1**, **Supplementary Figures 4C, D**). *CCL4, CCL5* and *CCL3L3* were the leading responsive genes in the chemokine module, while *NFKBIA* and *CD69* represented key nodes in inflammatory feedback and T cell activation, respectively.

Collectively, by integrating cell–cell communication profiling with in silico virtual knockout analysis, our data suggest that the CCL20-CCR6 axis may represent a cirrhosis-specific immune regulatory hub. Mechanistically, M2 macrophages are predicted to engage in bidirectional crosstalk with MAIT cells via CCL20-CCR6 signaling, and this interaction appears to be reinforced by a MAIT cell-autonomous autocrine loop, potentially forming a pathogenic positive feedback circuit. Intracellularly, CCL20 is predicted to function as a nodal regulator that may orchestrate core MAIT cell programs, including chemokine production, TCR/NK-like activation, NF-κB-mediated inflammatory signaling, and stress survival adaptation. This coordinated intercellular and intracellular regulatory network likely contributes to chronic hepatic inflammation, is associated with local immune cell retention and expansion, and is predicted to facilitate fibrotic microenvironment remodeling during cirrhosis progression.

### 3.7 Immunofluorescence validation of the cirrhosis-specific CCL20-CCR6 axis mediating MAIT-M2 macrophage interactions

**Figure 8:**
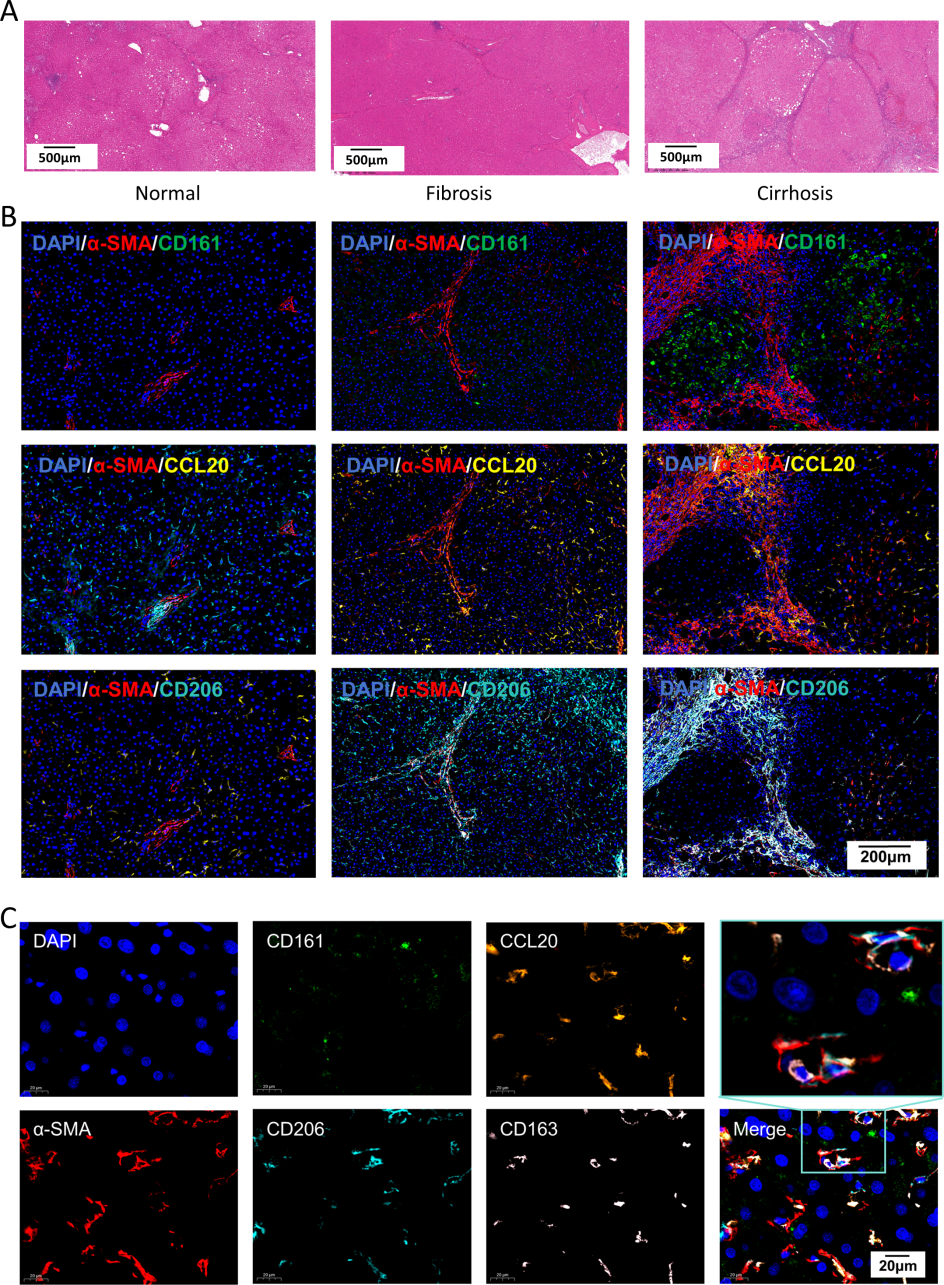
Immunofluorescence validation of CCL20-CCR6 axis-mediated MAIT-M2 macrophage interaction in liver cirrhosis. **(A)**. Hematoxylin and eosin (H&E) staining of normal, fibrotic, and cirrhotic liver tissues; **(B)** Quantitative analysis of co-expression fluorescence intensity of CD161, CCL20, CD206, and α-SMA; **(C)** Colocalization analysis of CCL20 with CD161 (MAIT cells) and CD206/CD163 (M2 macrophages) in cirrhotic liver tissue.

To visually validate the key molecular expression features and cellular interaction patterns identified through transcriptomic and single-cell analyses at the tissue level, immunofluorescence staining was performed on human liver tissue samples representing normal, fibrotic, and cirrhotic stages (**Figure 8A**). The analysis targeted CD161 (MAIT cells marker), CCL20 (core chemokine), CD206 (M2 macrophage marker), CD163 (M2 macrophage marker), and α-SMA (marker of activated hepatic stellate cells). Quantitative analysis revealed a progressive increase in the co-expression fluorescence intensity of CD161, CCL20, CD206, and α-SMA with disease progression (from normal to fibrosis to cirrhosis). Colocalization of these three markers with α-SMA was most pronounced in cirrhotic tissue, moderate in fibrotic tissue, and only weakly detectable in normal liver tissue (**Figure 8B**). Quantitative analysis of immunofluorescence staining (**Supplementary Material 1**, **Supplementary Figure 1D**) further confirmed these progressive changes: the percentages of CD161^+^, CCL20^+^ and CD206^+^ cells all increased significantly in a stepwise manner from normal to fibrosis and then to cirrhosis (CD161: P < 0.01, CCL20: P < 0.001, CD206: P < 0.01). These findings are highly consistent with transcriptomic data showing upregulation of core genes such as CCL20 during cirrhosis progression and with single-cell analyses indicating enriched activation of MAIT cells and M2 macrophages in cirrhosis. This concordance confirms that elevated expression of key molecules is closely associated with the pathological progression of liver fibrosis and cirrhosis.

Colocalization analysis demonstrated substantial overlap between CCL20 and CD161 in cirrhotic tissue, as well as strong colocalization between CCL20 and CD206/CD163 (**Figure 8C**). In addition, enhanced co-expression of CD161 and α-SMA was observed in the fibrotic septa of cirrhotic tissue, suggesting spatial proximity between MAIT cells and activated hepatic stellate cells within the pathological microenvironment and providing histological evidence for their potential functional interaction.

Together, these morphological findings directly confirm that CCL20 is specifically expressed in MAIT cells (CD161^+^) and M2 macrophages (CD206^+^/CD163^+^), with expression levels significantly increasing during cirrhosis progression. Furthermore, MAIT cells and M2 macrophages appear to form localized interactive units through CCL20 secretion and recognition, consistent with the bidirectional communication via the CCL20-CCR6 axis between these cell types predicted by single-cell transcriptomics. This tissue-level validation strengthens the pathological relevance and cellular source attribution of core molecules, offering direct morphological evidence supporting the role of the CCL20-CCR6 axis in driving immune dysregulation in liver cirrhosis.

## 4 Discussion

Cirrhosis represents a common end-stage manifestation of various chronic liver injuries, characterized by progressive fibrosis and vascular remodeling that ultimately lead to organ failure. Although diverse etiologies—including viral hepatitis, metabolic disorders, alcohol abuse, and cholestatic injury—contribute to cirrhosis development, accumulating evidence suggests that immune dysregulation constitutes a unifying and central mechanism in its pathogenesis and progression (37). In particular, sustained activation of inflammatory signaling cascades and inflammasome-mediated proinflammatory responses are widely acknowledged as core drivers of chronic liver disease progression. These inflammatory processes serve as critical links between immune dysregulation and progressive fibrogenesis, and their pathogenic roles across multiple chronic liver disease contexts have been systematically delineated in recent work (38). In this study, through integrative analysis of large-scale bulk and single-cell transcriptomic data, we propose and validate a novel immunoregulatory model: under cirrhotic conditions, MAIT cells and M2 macrophages establish a robust interactive network via the CCL20-CCR6 axis. MAIT cells may achieve self-regulation not only through intrinsic CCL20 expression but also by responding to CCL20 secreted by M2 macrophages. This mutual modulation, negligible in healthy livers, becomes markedly pronounced in the cirrhotic microenvironment. Our findings provide new insights into the reorganization of intercellular immune communication in cirrhosis and suggest that the CCL20-CCR6 pathway may serve as a potential therapeutic target for liver fibrosis and cirrhosis.

CCL20 is a potent chemokine whose only known receptor is CCR6. Previous studies have demonstrated robust induction of CCL20 in alcoholic hepatitis, viral hepatitis, and non-alcoholic steatohepatitis, with elevated hepatic and serum levels (15, 39, 40). Inhibition of CCL20 attenuates liver injury and profibrotic signaling (15). Of note, oxidative stress and lipid peroxidation, as core pathological processes of chronic liver injury, also act as important upstream triggers for chemokine upregulation and inflammatory cascade amplification (41). Mechanistically, visfatin induces macrophage *CCL20* expression via NF-κB and MKK3/6-p38 pathways, thereby promoting hepatic stellate cell activation and fibrosis marker expression (42). Our findings extend these observations by demonstrating that *CCL20* expression in MAIT cells dynamically increases along pseudotime trajectories and is significantly elevated in cirrhotic samples compared with healthy controls. Furthermore, the CCL20-CCR6 signaling axis shows specific transcriptional enrichment in MAIT cells and M2 macrophages in cirrhosis, indicating a disease-specific transcriptional activation pattern of this pathway.

Functional stratification of MAIT cells revealed that high *CCL20* expression was associated with increased cytotoxicity, progenitor exhaustion, and terminal exhaustion scores (P < 0.0001). This phenotype—characterized by preserved effector capacity alongside progressive exhaustion—suggests a maladaptive state typical of chronic inflammation. Autocrine CCL20-CCR6 signaling may transcriptionally support sustained MAIT cells activation-associated programs even in the absence of acute stimuli, thereby linking persistent inflammation to gradual immune dysfunction. Although direct causality remains to be established, the strong association between *CCL20* expression and exhaustion signatures suggests that this chemokine may serve as a functional bridge between chronic antigen exposure and immune failure.

To further explore the putative downstream pathways underlying this regulatory effect, we performed in silico virtual knockout analysis of *CCL20* in cirrhotic MAIT cells (**Supplementary Material 1**, **Supplementary Figure 4**). Transcriptional perturbation profiling showed that *CCL20* ablation induced significant upregulation of *NFKBIA*, a core negative feedback regulator of NF-κB signaling, implying that tonic CCL20-CCR6 signaling may sustain MAIT cell activation via persistent NF-κB pathway engagement, a signaling axis previously documented in multiple immune cell contexts. In parallel, virtual knockout upregulated a set of activation- and exhaustion-associated surface molecules (CD69, CD160) and secondary inflammatory chemokines (*CCL4, CCL5, CCL3L3*). Functional module enrichment further confirmed that *CCL20* perturbation predominantly affects biological programs including chemokine-mediated inflammatory recruitment, TCR/NK-like activation, and NF-κB immediate early signaling. These data support a working model whereby chronic *CCL20* stimulation may maintain persistent low-grade activation of MAIT cells through NF-κB-dependent signaling and secondary autocrine chemokine circuits; prolonged exposure to this activating milieu may progressively push cells toward a cytotoxic yet exhausted state, as frequently observed in chronic liver disease settings.

Macrophages exhibit substantial plasticity and play central roles in liver injury and repair. While classically activated M1 macrophages promote acute inflammation, M2-polarized macrophages contribute to tissue repair but can also secrete profibrotic mediators such as TGF-β and PDGF in chronic settings (43). Our cell-cell communication analysis indicates that M2 macrophages function as immunological hubs in cirrhosis by actively engaging MAIT cells through CCL20-CCR6 signaling. Notably, this interaction is largely restricted to M2 rather than M1 macrophages, underscoring functional specialization within the monocyte-macrophage lineage. Importantly, the M2-MAIT interaction is disease-specific. In healthy liver, CCL20-CCR6 signaling between these populations is minimal. In cirrhosis, however, M2-derived CCL20 may promote CCR6^+^ MAIT recruitment and activation, while MAIT cells reciprocally upregulate *CCL20*, potentially establishing a localized positive feedback loop. We hypothesize that microenvironmental factors such as persistent inflammatory signaling, hypoxia, and extracellular matrix remodeling promote this functional reprogramming of M2 macrophages. Sustained paracrine CCL20 stimulation may drive MAIT cells toward a cytotoxic yet progressively exhausted state, maintaining low-grade but chronic inflammatory output—a hallmark of immune imbalance in advanced liver disease.

Collectively, our data support a model in which chronic liver injury may induce persistent CCL20 production, leading to CCR6^+^ MAIT cells recruitment, retention, and autocrine reinforcement. This MAIT-M2 feedback circuit may amplify hepatic inflammation, sustain stellate cell activation, and promote extracellular matrix deposition, thereby accelerating fibrosis progression. These findings support the role of M2 macrophages in cirrhosis—not merely as fibrogenic effectors but as candidate orchestrators of pathogenic immune crosstalk.

Clinically, CCL20 levels in serum or liver tissue may serve as indicators of cirrhosis activity and progression risk. Given prior evidence linking CCL20 to inflammatory severity (15, 39, 40), our data further support its potential value in dynamic disease monitoring. Therapeutically, disruption of the CCL20-CCR6 axis—through neutralizing antibodies or CCR6 antagonists—has shown antifibrotic effects in preclinical studies (42, 44, 45). Targeted modulation of the MAIT-M2 interaction network may therefore represent a promising strategy for immune microenvironment-directed intervention in cirrhosis. Integrative in silico analytical frameworks of this kind have also been widely applied to mechanism exploration and candidate target screening for metabolic liver diseases, demonstrating robust research application value (46).

Despite the integrative multi-omics analyses and tissue-level validation performed in this study, several limitations should be acknowledged. First, the conclusions were primarily derived from publicly available transcriptomic datasets and computational inference analyses, which may introduce cohort heterogeneity and potential selection bias. Second, we only confirmed the correlative crosstalk of the CCL20-CCR6 axis between MAIT cells and M2 macrophages via bioinformatics and immunofluorescence, without *in vitro* cell experiments and *in vivo* animal intervention models to verify its causal role in cirrhosis progression. Third, the dynamic regulatory mechanisms underlying MAIT cells exhaustion and macrophage polarization during cirrhosis progression were not fully elucidated. Future mechanistic studies and prospective clinical investigations are warranted to further clarify the pathogenic and therapeutic significance of the CCL20-CCR6 axis in liver cirrhosis.

From a methodological perspective, recent advances in spatially resolved multi-omic and functional genomic technologies offer promising avenues to address these limitations and deepen mechanistic exploration in future studies. Spatial CITE-seq realizes cellular-resolution transcriptome and protein co-mapping in intact tissues (47), enabling *in situ* visualization of MAIT cells, M2 macrophages and CCL20-CCR6 axis colocalization in cirrhotic liver, and dissection of cell heterogeneity in the fibrotic microenvironment. The Perturb-DBiT platform supports large-scale spatial CRISPR screening with transcriptome profiling in native tissues (48), which can validate the causal role of CCL20/CCR6 in MAIT cells dynamics and macrophage crosstalk. Spatial tri-omic sequencing (joint profiling of chromatin accessibility, transcriptome and proteome) (49) will delineate the full regulatory cascade of the CCL20-CCR6 axis, and uncover upstream mechanisms of MAIT cells reprogramming and M2 macrophage polarization.

Future mechanistic studies and prospective clinical investigations, empowered by these cutting-edge spatial and functional genomic approaches, are warranted to further clarify the pathogenic and therapeutic significance of the CCL20-CCR6 axis in liver cirrhosis.

## 5 Conclusion

This study uncovers a key feature of immune dysregulation in liver cirrhosis: the CCL20-CCR6 axis is associated with a pathogenic interaction between MAIT cells and M2 macrophages. M2-derived CCL20 may engage CCR6 on MAIT cells, which is associated with their transition toward a transcriptional profile of heightened cytotoxic potential and exhaustion. This process, reinforced by an autocrine CCL20 loop in MAIT cells, is linked to amplified intrahepatic inflammation and may contribute to fibrosis progression. The CCL20-CCR6 axis thus emerges as a cirrhosis-specific marker of immune remodeling and a potential therapeutic target, with CCL20 levels also warranting consideration as a biomarker of disease activity.

## Data Availability

The datasets presented in this study can be found in online repositories. The names of the repository/repositories and accession number(s) can be found below: https://www.ncbi.nlm.nih.gov/, GSE49541 https://www.ncbi.nlm.nih.gov/, GSE164760 https://www.ncbi.nlm.nih.gov/, GSE14323 https://www.ncbi.nlm.nih.gov/, GSE181483.
