# Supplementary material for: CCL20-CCR6 axis mediates mucosal-associated invariant T-M2 macrophage crosstalk to drive immune dysregulation in liver cirrhosis
Source: Front Immunol. 2026 Jul 20;17:1859932. doi: 10.3389/fimmu.2026.1859932 (PMC13429398; doi:10.3389/fimmu.2026.1859932)
Supplement: Supplementary file 1 [file Supplementaryfile1.docx]

| Software Package | Version | Primary Application |
| --- | --- | --- |
| R (Base) | 4.4.1 | Runtime environment for all statistical analyses |
| Seurat | 5.1.0 | Single-cell transcriptomic data processing, clustering and visualization |
| Harmony | 1.2.0 | Single-cell transcriptome batch effect correction |
| Monocle | 2.32.0 | Pseudotime trajectory construction and analysis |
| CellChat | 1.6.1 | Cell-cell communication network inference |
| TCellSI | 1.2.0 | T cell functional state scoring |
| GSVA | 1.52.3 | Single-sample gene set enrichment analysis (ssGSEA) |
| limma | 3.60.3 | Bulk transcriptome differential expression analysis |
| sva | 3.54.0 | Batch effect correction for merged bulk datasets |
| glmnet | 4.1.8 | LASSO regression feature selection and model construction |
| WGCNA | 1.73 | Weighted gene co-expression network analysis |
| IOBR | 0.99.8 | Immune cell infiltration deconvolution |
| clusterProfiler | 4.12.0 | GO and KEGG functional enrichment analysis |
| ggplot2 | 3.5.1 | Statistical graphics and data visualization |
| scTenifoldKnk | 1.0.3 | Virtual gene knockout and gene regulatory network perturbation analysis |

Bulk differential expression analysis: Significance threshold set as adjusted P < 0.05 (Benjamini-Hochberg correction) with |log₂fold change| ≥ 0.25.

WGCNA: Top 5000 genes with the highest median absolute deviation (MAD) were selected for network construction; soft-thresholding power was determined by the scale-free topology criterion; minimum module size = 30; modules with correlation > 0.75 were merged.

LASSO regression: 10-fold cross-validation was applied; optimal penalty parameter λ was selected at lambda.min (minimum cross-validation classification error); alpha = 1 (L1 regularization).

Single-cell data preprocessing: Quality control thresholds: 200 < nFeature_RNA < 7000, percent.mt < 15%; 2000 highly variable genes retained; batch correction performed with Harmony using the first 40 principal components.

Pseudotime analysis: DDRTree algorithm for dimensionality reduction (max components = 2); trajectory root state was assigned based on the memory T cell subtype.

Cell-cell communication analysis: Performed based on the CellChatDB.human ligand-receptor database; cell groups with fewer than 10 cells were filtered out; significance calculated via built-in permutation test.

ssGSEA scoring: Enrichment scores calculated via the ssGSEA algorithm in the GSVA package with default settings.

Virtual knockout analysis: Performed using the scTenifoldKnk package on MAIT cells from cirrhotic samples; input gene set comprised the target gene *CCL20* and 3000 highly variable genes selected by the vst method; quality control thresholds: maximum mitochondrial gene ratio = 0.1, minimum library size = 500; 10 subnetworks constructed with 500 randomly sampled cells per network; statistical significance determined by built-in chi-square test with Benjamini–Hochberg correction.


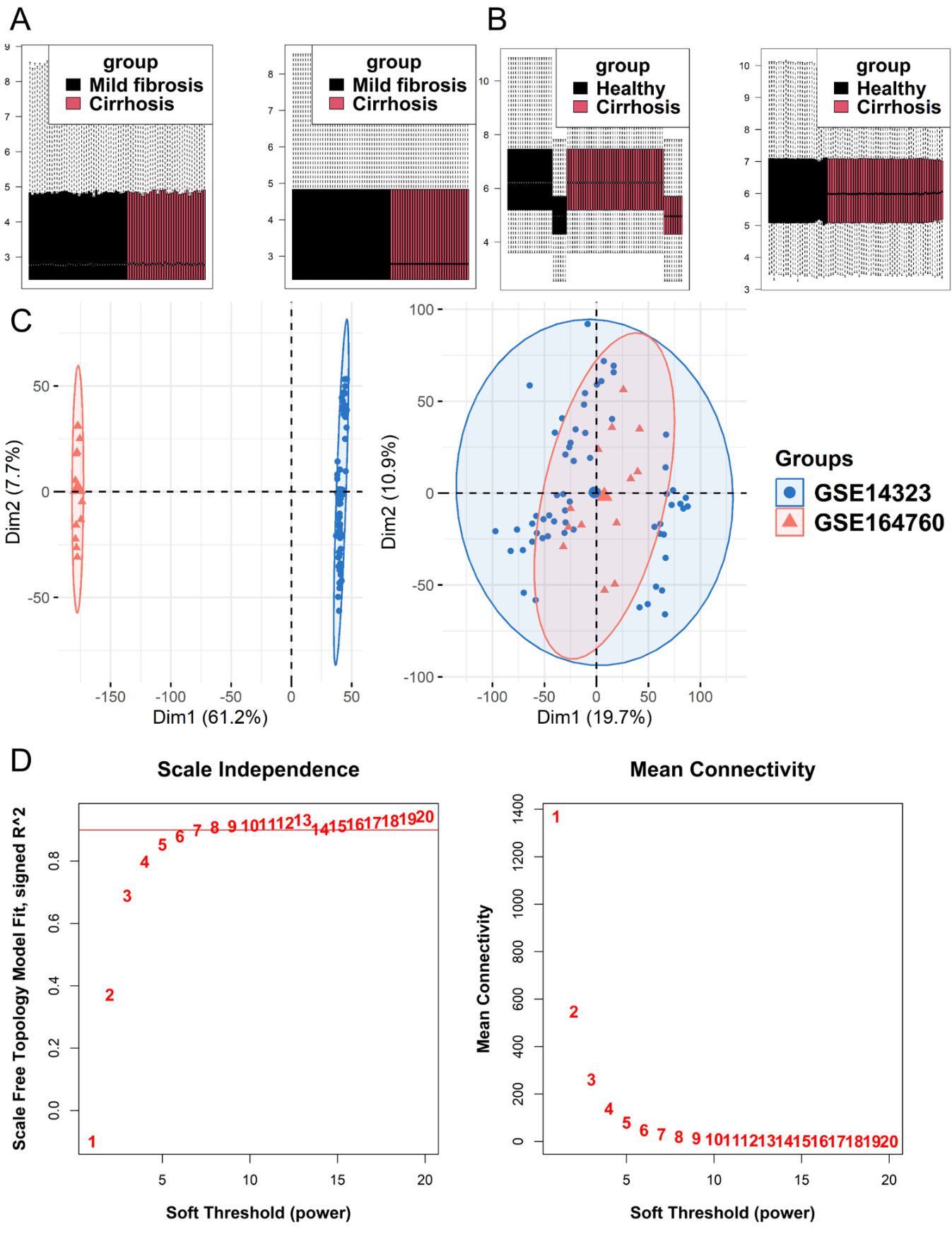


Figure 1

A. Normalization of gene expression data from dataset GSE49541

B. Batch effect correction using the SVA package for the merged dataset (GSE14323 and GSE164760)

C. Principal Component Analysis (PCA) of the merged dataset (GSE14323 and GSE164760) before and after batch effect correction

D. Determination of the optimal soft-thresholding power for Weighted Gene Co-expression Network Analysis (WGCNA)


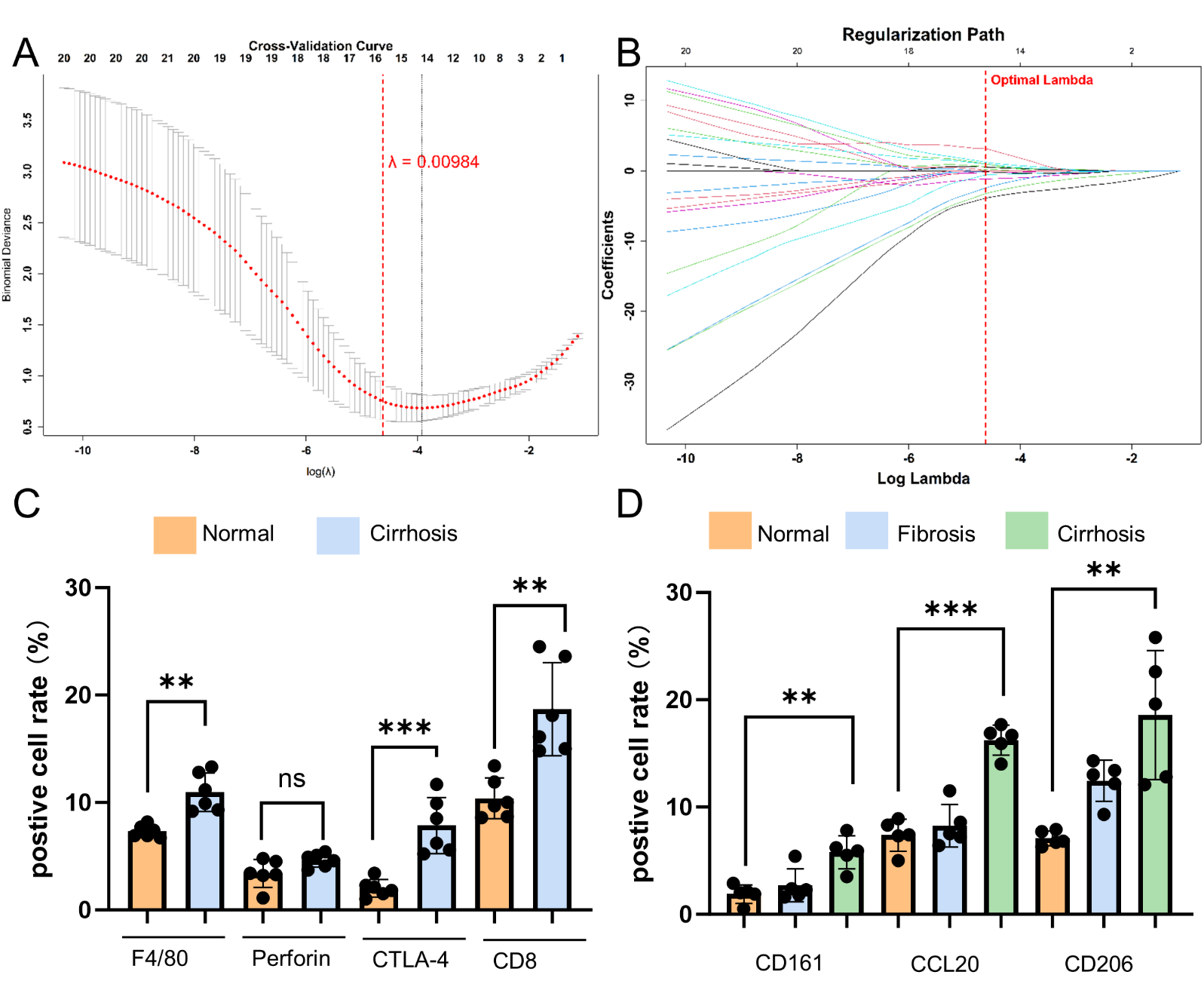


Figure 2

A. Cross-validation curve for LASSO regression model selection

B. Regularization path of the LASSO regression model

C. Quantitative analysis of immune marker positive cell rates in normal and cirrhotic liver tissues

D. Quantitative analysis of immune marker positive cell rates in normal, fibrotic and cirrhotic liver tissues


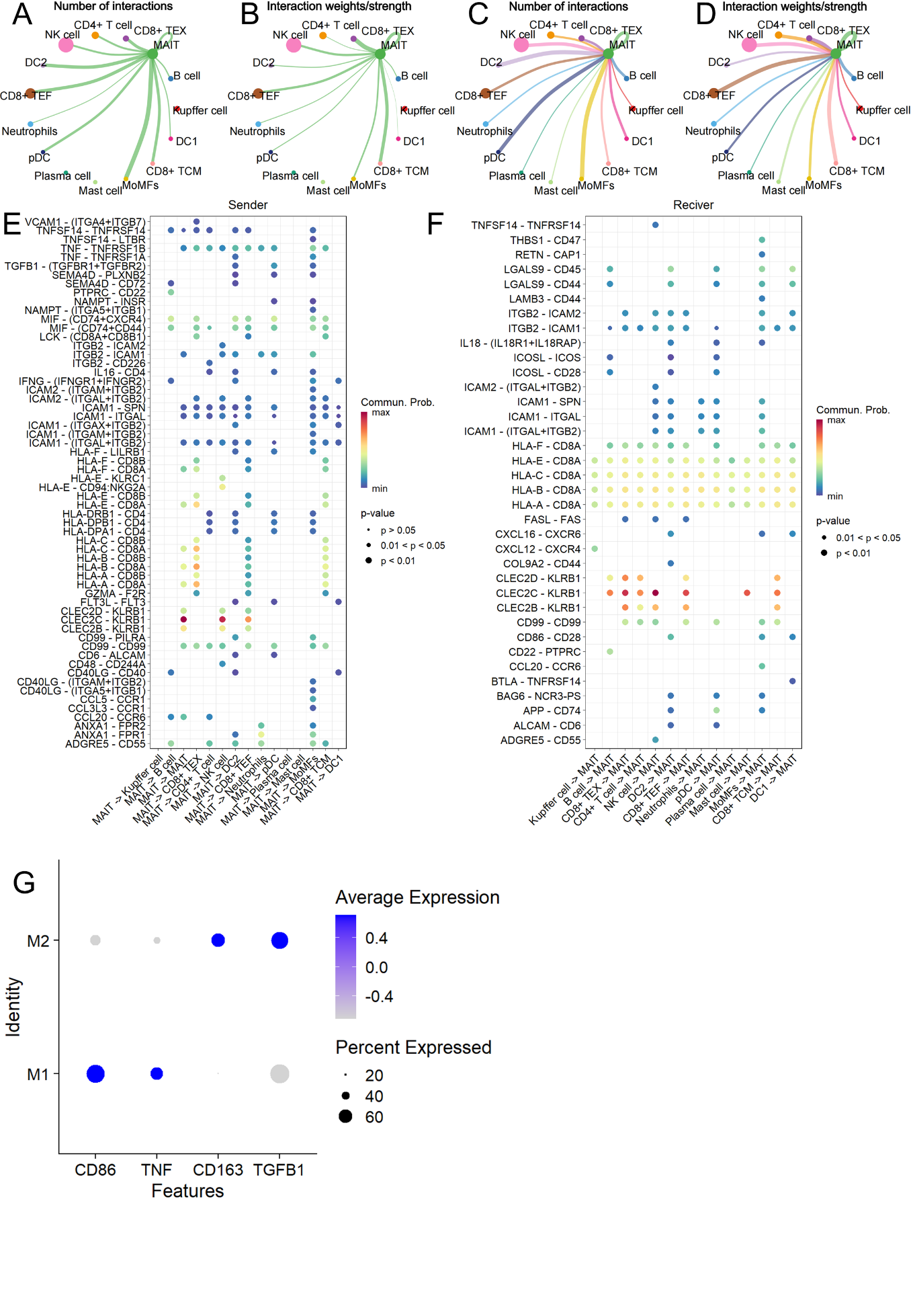


Figure 3

A-D. Network visualization of the number and strength of immune cell-cell interactions in cirrhotic samples;

E-F. Bubble plots showing communication probabilities of ligand-receptor pairs associated with all signaling pathways in MAIT cells

G. Bubble plot visualization of M1 and M2 macrophage subset marker expression


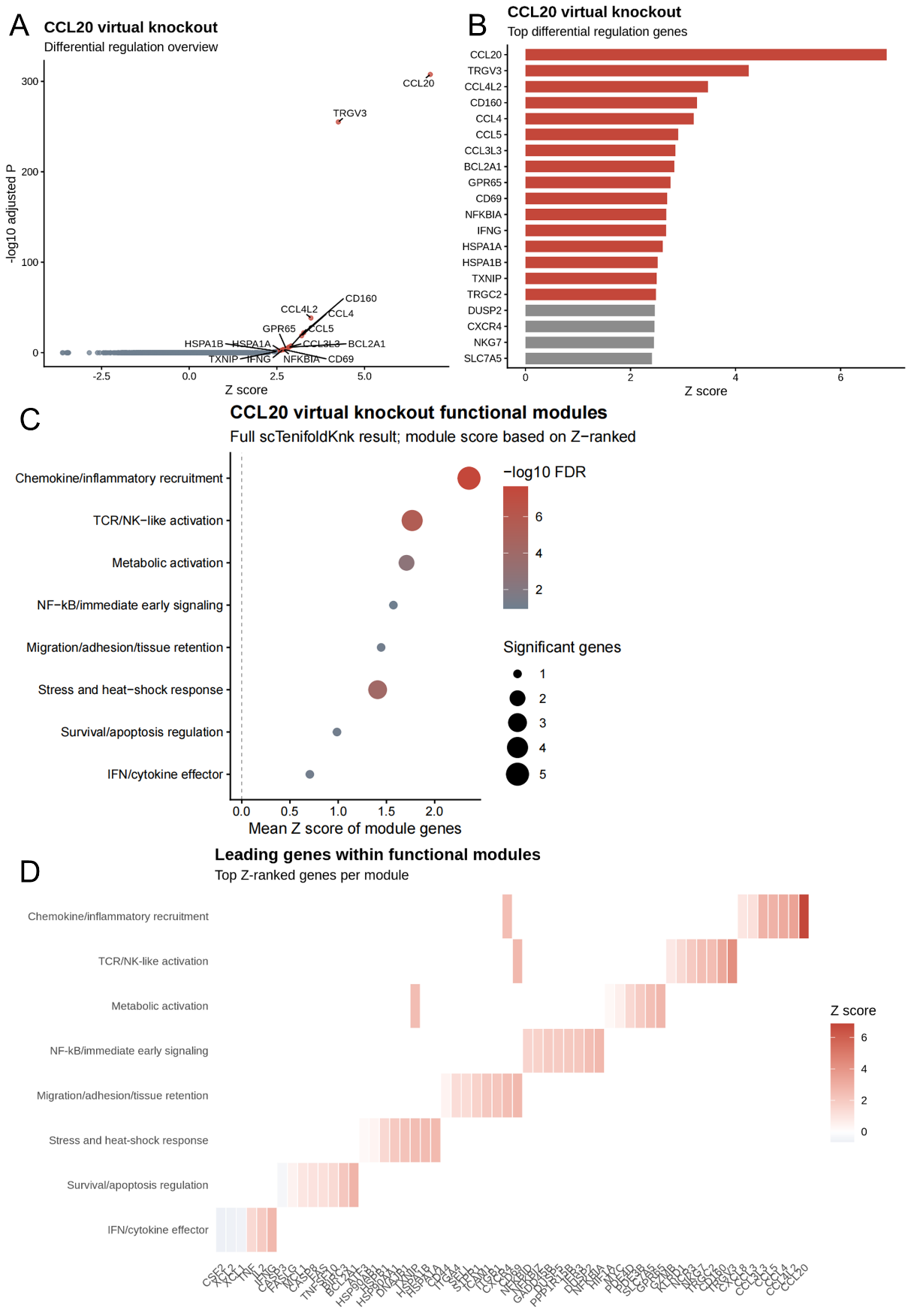


Figure 4 Transcriptomic differential regulation and functional module annotation of CCL20 virtual knockout.

A.Volcano plot showing the overview of differentially regulated genes. The x-axis represents the Z-score, and the y-axis indicates the statistical significance (-log10 adjusted P-value). Red dots indicate genes with significant differential expression(adjust P < 0.05).

B. Bar plot displaying the top 20 genes with the highest Z-score ranking. The x-axis represents the Z-score, and the y-axis lists the gene names. All the displayed genes exhibit positive Z-scores (upregulation). Red bars represent statistically significant genes, whereas gray bars denote non-significant genes.

C.Bubble plot illustrating the functional modules enriched upon CCL20 virtual knockout based on Z-score ranking. The x-axis shows the mean Z-score of genes within each module. Bubble size indicates the number of significant genes per module, and the color gradient reflects the -log10 FDR, with darker red representing higher statistical significance.

D.Heatmap depicting the leading genes (top Z-ranked genes) within each functional module. The color scale represents the Z-score, with darker red indicating stronger regulation of the corresponding genes.
